# A systematic review of minimum important changes for generic multi-attribute utility instruments and recommendations for their estimation

**DOI:** 10.1007/s10198-025-01778-3

**Published:** 2025-04-16

**Authors:** Glen J. Henson, Ingrid van der Mei, Bruce V. Taylor, Paul Scuffham, Gang Chen, Julie A. Campbell

**Affiliations:** 1 Menzies Institute for Medical Research (University of Tasmania), 17 Liverpool St, Hobart, TAS 7000 Australia; 2 Menzies Health Institute Queensland (Griffith University), G40 Griffith Health Centre, Level 8.86 Gold Coast Campus Griffith University, Southport, QLD 4215 Australia; 3 University of Melbourne, Parkville, VIC 3052 Australia

**Keywords:** Minimum important changes, Multi-attribute utility instruments, Health state utility, Health-related quality of life, Systematic review, Mathematic methods, C69 other mathematical methods, I18 public health

## Abstract

**Introduction:**

Minimum important changes (MICs) represent thresholds for clinically meaningful change. Multi-attribute utility instruments (MAUIs) generate health state utilities (holistic measures of health-related quality of life). No systematic review of MICs specifically for MAUIs has been conducted. In addition, no guidelines for estimating MICs for MAUIs have been proposed. We aimed to correct these evidence gaps by producing guidelines contextualised by a systematic review.

**Methods:**

We searched ten databases for relevant records using various search terms. Extracted data were analysed narratively and descriptively. The presence of key reporting items (relating to precision, sensitivity, and concurrent validity) was also evaluated. Guidelines for MIC estimation were informed by the broader MIC literature and contextualised using study results.

**Results:**

The review identified 5035 non-duplicate records, with 68 entering the study. 282 unique, anchor-based MICs were extracted. Of these MICs, 119 (42.20%) pertained to the EQ-5D-3L, 82 (29.08%) to the EQ-5D-5L, and 50 (17.73%) to the SF-6D.v1. The most common anchor-based method used to estimate MICs (107, 37.94%) involved taking the mean change score for a group considered to have experienced a MIC. Distribution-based methods were also common, appearing in 31 (45.59%) of the included studies. The inclusion of key reporting items was generally deficient.

**Conclusions:**

Deficiencies in reporting and diverse estimation methods raise concerns regarding the extant MAUI MIC literature. Researchers should exercise caution when using existing MAUI MICs. Recommendations presented in our study may assist researchers in effectively estimating MICs for use in health economics.

**Supplementary Information:**

The online version contains supplementary material available at 10.1007/s10198-025-01778-3.

## Introduction

### Multi-attribute utility instruments

Multi-attribute utility instruments (MAUIs) are commonly used to measure health-related quality of life [1]. MAUIs comprise questionnaires that describe potential health profiles (described as health states) and value-sets that represent societal preferences in these health profiles. For any health state, a MAUI can generate a health state utility. This health state utility is a health-related quality of life metric and represents the relative position (and therefore desirability) of a person's health state on an interval scale anchored at zero (equivalent to death) and one (equivalent to full health) [2]. Some instruments can generate negative health state utilities, which are regarded as being worse than death. Health state utilities are assigned to health states using a variety of economics techniques, including the standard gamble, visual analogue scale, time-trade off, and relative social willingness to pay, in conjunction with multi-attribute utility functions [3]. Health state utilities are frequently applied in cost-utility analyses (a type of comprehensive health economic analysis, used to evaluate medical interventions), clinical assessments, and evaluations of patient-reported outcomes [4, 5].

### Minimum important changes

Similar to other patient-reported outcome measures, understanding what constitutes a clinically meaningful change in health state utility is essential to the effective usage of MAUIs [6]. To determine the magnitude of a clinically meaningful change, researchers must obtain estimates of the minimum important change (MIC). In this study, we define a MIC as:

The minimum, within-person change in score (positive or negative) on a patient-reported outcome measure that informed participants (or proxies) perceive as important, given their personal situations and characteristics, and would lead a patient, clinician or other concerned party to consider a change in an individual’s disease management or treatment.

To the best of our knowledge, no guidelines exist to inform effective and transparent estimation of MICs for MAUIs.

### Aims of the systematic review

The principle aim of our systematic review was to summarise and synthesise all extant literature relating to MICs for MAUIs. The results of our review were to inform the attainment of our secondary aim, which comprised the compilation of guidelines and recommendations for the effective estimation of MICs, in the context of existing estimates. The ordering of these aims corresponds with the methodological recommendations of the Professional Society for Health Economics and Outcomes Research Good Research Practices Task Force report regarding health state utilities in clinical studies, which states that “a review of … the published literature is an important starting point to determine whether [relevant] high-quality estimates … are available and whether new are needed” [7].

Health state utility is a useful outcome to include in clinical trials as it constitutes a holistic, scalable, and universal measure of health-related quality of life and can be used to evaluate the cost-utility of trialled interventions, which can inform pharmaceutical subsidisation policies. The findings of this study may assist researchers in establishing reliable endpoints for studies that use health state utilities to determine participant outcomes. In turn, may encourage the inclusion of MAUIs in a wider range of studies, including clinical trials.

## Methods

This review was registered with PROSPERO (registration number CRD42021261821). A detailed report of the methods of this review is available in our published protocol [8] (accessible free of charge), which adhered to the Preferred Reporting Items for Systematic Reviews and Meta-Analyses Protocols guidelines (PRISMA-P) [9]. This study followed the PRISMA reporting guidelines and the Consolidated Health Economic Evaluation Reporting Standards (applied in the results sections) [10, 11]. It was also informed by the Professional Society for Health Economics and Outcomes Research Good Research Practices Task Force report regarding health state utilities in clinical studies, as stated above [7].

### Minimum important change terminology and definitions

Many terms and definitions of MIC (minimum important change) have been proposed since the publication of the seminal paper by Jaeschke et al*.* in 1989 [12, 13]. Table 1A provides two MIC definitions, sourced from the broader literature, as well as the insights we gained from those that were incorporated into our own definition. This definition, presented above in the "Minimum important changes" section, informed analysis and discussion in our systematic review by establishing an expectation regarding what a MIC should be.

**Table 1:** Anchor-based minimum important change (MIC) definitions and credibility appraisal criteria

Key messages	Definition
*A. MIC definitions*	
MICs must represent the *smallest*, meaningful difference Participants must understand their role in MIC determination MICs should be able to inform meaningful decisions regarding clinical practice^a^	“The smallest difference in score in the outcome of interest that informed patients or informed proxies perceive as important, either beneficial or harmful, and which would lead the patient or clinician to consider a change in the [patient’s] management”, Schunemann and Guyatt (2005) [16]
Patient perspectives must have primacy in MIC estimation MIC values may be contingent on population and study characteristics	“A change [in an outcome measure] that patients would consider important to reach in their situation, dependent on baseline values or severity of disease, on the type of intervention and the duration of the follow-up period”, De Vet et al*.* (2006), as reproduced by King et al. (2011) [12, 17]
Our definition also acknowledges the United States’ Food and Drug Administration recommendation that MICs should focus on individual-level/within-person change	“The minimum, within-person change in score (positive or negative) on a patient-reported outcome measure that informed participants (or proxies) perceive as important, given their personal situations and characteristics, and would lead a patient, clinician or other concerned party to consider a change in an individual’s disease management or treatment”, definition provided in our study

Item	Explanation
*B. Credibility appraisal criteria*	
1. Was the correlation between the instrument and anchor measured?	A valid anchor must be closely related, and thus at least moderately correlated, with the relevant instrument
2. Was the sensitivity and specificity of the MICs evaluated?	The efficacy of a MIC is best determined by how effectively it can separate those defined as experiencing at least a MIC in score from those who have not. Therefore, we consider the reporting of sensitivity and specificity as necessary in studies estimating MICs
3. Was the precision of the MICs reported?	Standard errors (SEs) or an equivalent measure (such as interquartile range) must be reported for MIC estimates. Note that SEs can be estimated in all scenarios, if not directly then by simulation (for example, by bootstrapping SEs)
4. Did the MIC estimate represent a small but meaningful difference?	Some studies define MICs using participants reporting large changes on, for instance, global rating of change scales. These studies misreport their estimates, as they do not necessarily constitute *minimum* important changes
5. Was the anchor appropriate for use with the study population?	Anchor questions must be relevant to the study population. Any MIC estimated using an anchor that does not meet this requirement is invalid

^a^This point is supported by several other definitions, though with a noticeable omission of reference to patient decision-making [13, 18–20]. Contradictorily, a recent editorial in *Quality of Life Research* did not support this view [21]. In response to this, we suggest that a MIC, the attainment of which has no clinical relevance, cannot be a reasonable object of study as it lacks an empirical implication

To establish our preferred terminology, and thus facilitate our discussions, we utilised the following criteria. Regarding the first word, we selected “minimum” over “minimal” or “minimally” as the former means “the least or smallest amount or quantity possible, attainable, or required”, whereas the latter is synonymous with negligible. We used “important”, rather than “significant” or “detectable”, to differentiate the MIC from purely statistical measures. Meaningful, and perhaps other, similar words could be substituted for important; however, important is used regularly in the literature and so was preferred. Lastly, we employ “change” as it is non-specific regarding direction (unlike “improvement” or “decrease”) and implies longitudinal within-person, rather than cross-sectional between person/group “difference” [14]. The United States Food and Drug Administration guidelines support that MICs should focus on individual-level change [15].

### Inclusion criteria

Studies eligible for inclusion in this review were any English language study that estimated a MIC (directly expressed in health-state utility) for a MAUI. Studies were ineligible if they were published before 1989 (the year that MICs were introduced in the literature and described as minimal clinically important differences) [13]. Case, in vitro, and animal studies were also ineligible. Furthermore, only original, peer-reviewed works were included. Hence, published abstracts, editorials, commentaries, protocols, reviews, meta-analyses, and unpublished works were ineligible.

### Search strategy and data extraction

Over the seven days ending 7 June 2022, we interrogated a variety of biomedical and economic databases, including Medline and Embase. The specifics of our comprehensive search strategy are presented in Appendix A. Using software (Covidence, 2022), studies were independently screened by two authors (GJH and JAC) based on the contents of their titles and abstracts. Full-text review of selected articles followed (using EndNote) and was conducted independently by the same two authors. Data extraction was undertaken principally by GJH, following a data extraction form, with twenty per cent of the data collection reviewed for accuracy by JAC.

The following groups of variables were sought for extraction: *Participant Characteristics*, including age, sex, number of participants, socioeconomic status, education level, health insurance coverage, exposure to socialised medicine, medication/intervention usage, diseases and comorbidities, country of interest, attrition, and response rate; *Publication Attributes*, including first and last author, date and journal of publication, country of origin, study type/design, and adherence to validated guidelines; *Mathematic Features*, including instruments of interest, methods of MIC calculation, direction (improvement, deterioration, or bidirectional) of MIC, and approach to MIC evaluation; *Sample Selection*, including exclusion and inclusion criteria, participant recruitment methods; *Results*, including MIC values, standard errors, measures of statistical robustness (such as standard error of measurement and minimum detectable change), and key discussions. Notably, seven corresponding authors were contacted to clarify information extracted from included studies, with one electing to respond.

### Quality appraisal: studies

The included studies were assessed for their credibility. Following a thorough examination of more recent literature we concluded that this study should deviate from the published protocol regarding credibility assessment. We adapted the core items of an instrument developed by Devji et al. [19] for evaluating the credibility of anchor-based MICs. This instrument has been applied in a similar and recent review of anchor-based MICs [22]. The credibility of distribution-based and instrument-defined MICs was not assessed. This is because, unlike anchor-based MICs, they rely only upon the correct execution of calculations. Consequently, credibility criteria are not applicable to them.

The criteria of our credibility appraisal are listed in Table 1B. Adaption of the original system was necessary as we wished to evaluate the credibility of studies based on a standard of reporting, rather than evaluate the quality of the estimated MICs. Also, some of the original instrument’s items were not relevant in our analysis, such as “Is the patient or necessary proxy responding directly to both the PROM and the anchor?” and “Is the anchor easily understandable and relevant for patients or necessary proxy?”. We also elected to add additional items based on observations made during our investigation. For example, we considered the reporting of sensitivity and specificity to be important when estimating anchor-based MICs.

Based on the percentage of criteria met, studies were rated manually by GJH and JAC as having low, medium, high, or very high *informativeness* depending on whether their percentage score was ≤ 0.25, 0.26–0.50, 0.51–0.75, or > 0.75. Percentage calculation denominators were reduced in the case of non-applicable items. Item five was excluded as all studies met this criterion; unless the included literature demonstrates failure on this item, we recommend its exclusion from future studies due to redundancy. Importantly, percentage scores were intended only to be indicative of the percentage of relevant reporting items included in an article, not a measure of an article’s scientific validity.

### Quality appraisal: body of evidence

To evaluate the risk of bias in our systematic review’s body of evidence, we used the Risk of Bias assessment tool for Systematic reviews (ROBIS) [23]. We deviated from the protocol in not implementing the Grading of Recommendations, Assessment, Development and Evaluations (GRADE) framework [24]. This was because the GRADE framework was inappropriate for our study, being designed for systematic reviews of evidence specifically pertaining to medical interventions.

### Data analysis

#### Descriptive and subgroup analyses

Summary data from extracted studies are presented as frequency and percentage and median and interquartile range, where appropriate. Frequencies and percentages were used to summarise study and MIC characteristics. The median and interquartile range were used to summarise quantitative MIC attributes, as well as the values of MIC estimates overall and by subgroup. MIC subgroups were defined according to estimation methodology, instrument, and direction of effect.

#### Qualitative analysis

As noted above in the "Search strategy and data extraction" section, the details of MIC estimation were extracted from each study and analysed thematically. Our inductive analysis focused on paradigms of different estimation techniques extracted from included studies [25]. Where appropriate, additional references from the broader literature were sourced to enhance our descriptions.

#### Data restrictions

Due to limitations in the data, our analysis was reduced in scope from what was outlined in the protocol. In particular, meta-analysis and meta-regression were excluded principally due to the presence of multiple MICs in many studies, which could not be pooled. Meta-analyses were also made difficult because of a lack of standard error data.

Furthermore, MICs estimated in the context of major medical interventions (such as surgery) were excluded from MIC stratifications. This was because they were both systematically larger than other MICs and representative of the changes participants expected from a particular intervention, rather than being of a general nature. These MICs were not excluded from the study and MIC characteristic summaries, however, as these were intended to describe the data we extracted.

## Results

### Search results

**Fig. 1:**
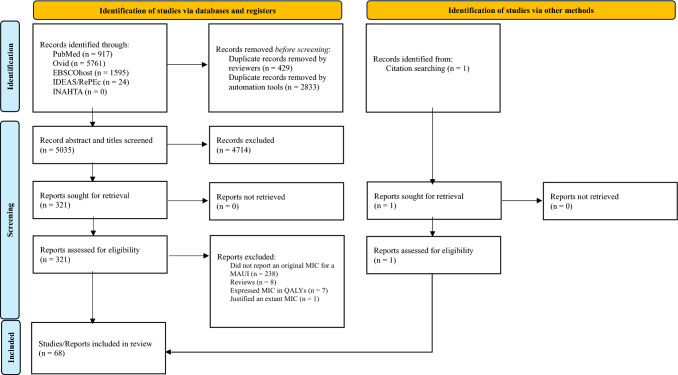
PRISMA flow diagram of studies eligible for inclusion in the systematic review

Database search and screening results are summarised in a PRISMA flow diagram labelled Fig. 1. This review identified 8297 records, 3262 of which were duplicates. Of the 5035 records screened, 321 met the eligibility criteria. All 321 reports were retrieved successfully. The major cause of ineligibility at this stage was irrelevance identifiable exclusively through full-text review. Additionally, seven studies that ostensibly met the inclusion criteria were excluded as they presented MIC estimates in quality-adjusted life years, rather than health state utility points [26–32]. Overall, 67 studies identified by our search strategy entered the review [14, 33–98], with an additional study being identified during citation searching and subsequently included [99]. From the total 68 accepted studies 282 unique anchor-based and instrument-defined MICs were obtained. Distribution-based MICs were not extracted as they are approximations only and do not constitute current best practice [100].

**Fig. 2:**
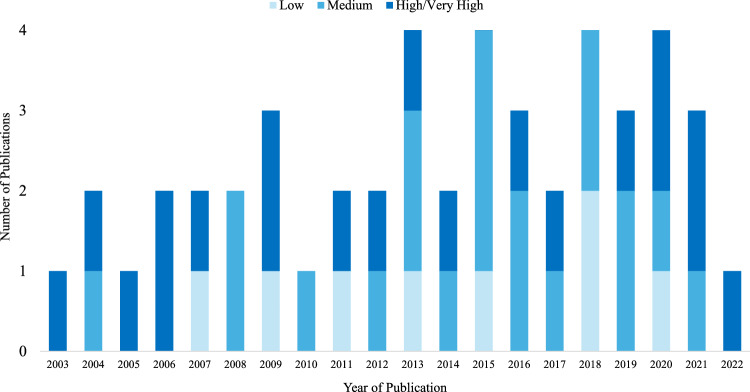
Column chart representing years of publication and reporting informativeness for eligible studies. *Notes* The percentages represented above only indicate the number of credibility criteria met by eligible studies; they do not indicate scientific validity. Also, this figure does not represent studies that produced only distribution-based or instrument-defined MICs. Publication of these excluded studies occurred as follows: 1 in 2005, 2010, and 2014; 2 in 2015, 2016, 2017, and 2019; 3 in 2020

The key characteristics of each included study are summarised in Supplementary Table 1, and the key characteristics of each MIC are summarised in Supplementary Table 2. These study characteristics are summarised below in the "Summary of study characteristics" section. The years of publication for the included studies are summarised in Fig. 2. As demonstrated by the figure, most studies were published after 2012 and the inclusion of reporting items was not observed to vary substantively over time.

### Results of data extraction

Data was available for most of the variables sought for extraction. Excluding reporting items examined during quality appraisal (see the "Summary of study characteristics" section below), data was complete for all included variables. Variables for which data was not available were socioeconomic status, education, health insurance coverage, exposure to socialised medicine, adherence to validated guidelines, and approach to MIC evaluation. Attrition and response rates were not collected due to differences between studies (i.e. data collection method, scope, study design) that made these figures meaningless for comparison and systematic quality appraisal. Moreover, details regarding participant recruitment were excluded as irrelevant; most studies involved valid multi-centre participant recruitment.

## Summary of study characteristics

Table 2 (supported by Supplementary Table 1) summarises the characteristics of the 68 included studies (note that multiple categories relating to the same characteristic can apply to each study). The most commonly used method of MIC calculation was the calculation of an average score attributable to individuals reporting or otherwise indicated to have experienced a MIC, according to the chosen anchor (41, 60.29%). This mean-based method was followed in prevalence by distribution-based standard deviation methods, which multiply the standard deviation in baseline health state utility scores by a constant (often between 0.5 and 0.2, inclusive) to estimate a MIC (28, 41.18%). The use of the receiver operating characteristic (ROC) curve was also prevalent (21, 20.88%). These estimation methods, and others presented in Table 2, are explained in detail in Table 3.

**Table 2:** Summary of the characteristics of the 68 included studies

Characteristic	Number	Percentage
Instrument		
EQ-5D-3L	44	64.71
EQ-5D-5L	15	22.06
SF-6D.v1	19	27.94
Quality of well-being	4	5.88
Health Utilities Index 2	4	5.88
Health Utilities Index 3	3	4.41
15-Dimension	3	4.41
Assessment of Quality of Life—4D	1	1.47
Method		
Anchor-based	55	80.88
Mean	41	60.29
Difference of means	9	13.24
Median	1	1.47
Percentile	2	2.94
Equipercentile	1	1.47
ROC curve^a^	21	30.88
Regression	12	17.65
Distribution-based	31	45.59
Standard deviation	28	41.18
Minimum detectable change	3	4.41
Standard error of measurement	8	11.76
Instrument-defined	8	11.76
Quality appraisal item^b^		
Correlation	34	61.82
Sensitivity and specificity	22	40.00
Precision	33	60.00
Small but meaningful difference	40	72.00
Overall quality		
Equal to 0%	2	3.64
Less than or equal to 25%	6	10.91
Less than or equal to 50%	22	40.00
Less than or equal to 75%	19	34.55
Greater than 75%	6	10.91

^a^ROC is an abbreviation of Receiver Operating Characteristics

^b^The following categories refer to the first four items in represented in Table 2, which were: (1) “Was the correlation between the instrument and anchor measured?”, (2) “Was the sensitivity and specificity of the MICs evaluated?”, (3) “Was the precision of the MICs reported?”, and (4) " Did the MIC estimate represent a small but meaningful difference?

**Table 3:** Methods of MIC estimation extracted from the included studies

	Anchor-based methods
*Estimation by means*	The study by Bilbao et al. [35] exemplifies this method. This study used a five-level global rating of change item as an anchor to estimate a MIC for the EQ-5D-5L. This item asked participants to evaluate the change in their health between baseline and follow-up, with level three indicating no change, levels two and four minor change, and levels one and five major change in the positive and negative directions. The study classified participants selecting levels two and four as experiencing minimum but meaningful change in their health-related quality of life. MICs were estimated by calculating the mean change in health state utility (HSU) scores, between baseline and follow-up, for these participants
*Estimation by difference of means*	This technique was used by Hoehle et al. [47]. As above, this study also utilised a five-level global rating of change item to estimate a MIC for the EQ-5D-5L. Their MIC for improvement was calculated as the difference in the mean change in HSU score for the groups of participants reporting levels two (“a little better”) and three (“about the same”) and comprised the difference between them. The purpose of the subtraction is to adjust for potential bias in ratings, with the resulting MIC representing the difference in the change in HSU scores necessary for participants to rate themselves as meaningfully improved, as opposed to not [101]
*Estimation by medians*	This method was only employed in one report that entered this review, authored by Gandhi et al. [40]. It is analogous to estimation by means except that medians are calculated instead. The use of the median in the referenced paper was based on the view that its associated inter-quartile range provided a superior measure of precision compared to the standard error, due to the elimination of parametric assumptions
*Estimation by equipercentile equating*	Estimation by equipercentile equating is a very novel technique, used in only one eligible study [87]. Equipercentile equating seeks to find the cumulative percentage associated with a particular score on one scale and equate this to a score on another scale with the same percentile rank [102]. For example, if 30% of participants reported a small but meaningful improvement or less on an anchor scale, this would be equipercentile equated to the change in HSU at the 30th percentile rank
*Estimation by percentiles*	This method involves estimating a cumulative percentage curve as a function of the change in HSU score among participants who reported small but meaningful changes in their health (on a global rating of change scale, for example) [103]. The curve can be conceived of as existing on a graph with the cumulative percentage of participants on the y-axis and change in HSU on the x-axis. The coordinate at which the curve flattens denotes the MIC value that has relatively high sensitivity but does not excessively reduce specificity to capture
*Estimation by ROC curve*	The receiver operating characteristics (ROC) curve estimates sensitivity (the proportion of truly assigned positives) and specificity (the proportion of truly assigned negatives) of all possible MICs and maps them on a chart with sensitivity represented on the y-axis and specificity on the x-axis. A MIC may then be selected based on one of several mathematical rules. One rule maximises the sum of sensitivity and specificity (sensitivity + specificity − 1, Youden’s Index), whereas another finds a MIC such that the resulting coordinate of the receiver operating characteristic curve is closest to the point (0,1) at which there is perfect sensitivity and specificity [(1 − specificity)^2^ + 1 − sensitivity)^2^] [14, 104]. A regression-based, parametric version of this method was utilised by Lamu et al. [60, 105]
*Estimation by regression*	Estimation by regression is often useful in the context of auxiliary instruments. For example, Nolan et al. [76] utilised the Chronic Respiratory Questionnaire as an anchor, which has a preestablished MIC of ten. Regression was conducted to determine the association between a one-point change on the Chronic Respiratory Questionnaire and change in EQ-5D-5L HSU. The resulting coefficient was multiplied by ten, thus providing a MIC of 0.059. Regression techniques have also been applied to control for baseline HSU scores (and consequently regression to the mean; see below), as in Most et al. [72]

	Distribution-based methods
*Approximation by standard deviation*	This distribution-based method was originally popularised by Norman et al. [106]. in their seminal paper, “Interpretation of changes in health-related quality of life: the remarkable universality of half a standard deviation” As the name suggests, approximation by standard deviation involves multiplying the baseline standard error (SE) of HSU scores by a constant. Based on Cohen’s guidelines regarding effect sizes [107], subsequent studies have used constants between 0.2 and 0.5 (inclusive) in the multiplication [24, 61, 63]
*Approximation by SEM or MDC*	As explained by Wyrwich et al. (2024) “The [standard error of measurement] SEM is the standard error in an observed score related to measuring with a particular test that obscures the true score”, and is calculated as SE_Baseline_ × (1 − r)^0.5^, where *r* is a reliability coefficient (often either a test–retest or intraclass correlation statistic) for a MAUI [73, 108]. Notably, when the reliability coefficient is 0.75, the aforementioned equation reduces to SE_Baseline_ × 0.5. The minimum detectable change (MDC) represents “the smallest change in score that can be detected beyond random error” [109], and is a function of the standard error of measurement being calculated as SEM × 1.96 × 2^0.5^, where 1.96 is the z-score associated with a 95% level of confidence [90]
*Instrument-defined estimation*	This technique is exemplified by McClure et al. (2017) in which MICs are estimated for the EQ-5D-5L [69]. Concisely, this method involves calculating the average, absolute differences in HSU between adjacent health states for any given instrument. Drawing on the cited study, an example of adjacent health states in the EQ-5D-5L are 33333 (reporting level three for all five items) and 33332 (reporting level three for all items except item five, for which level two is reported)

Table 2 also shows that the most prevalent instrument for which MICs were estimated was the EQ-5D-3L (*n* = 44, 64.71%), followed by the SF-6D.v1 (*n* = 19, 27.94%), and the EQ-5D-5L (*n* = 15, 22.06%). Of the eligible studies that used anchor-based methodologies, 61.82% (*n* = 34) calculated correlations between the instrument and anchor (establishing construct validity), 40.00% (*n* = 22) estimated specificity and sensitivity as a diagnostic for MIC effectiveness, 60.00% (*n* = 33) determined the precision of their estimates, and 72.00% (*n* = 40) had an anchor that measured a small but meaningful difference in quality of life. Based on our criteria, 45.46% (25 of 55) of studies were ranked as being of high informativity.

Additionally, for anchor-based methods, a global rating of change item (or equivalent) was the most common type of anchor, with such items most frequently having five levels (27 of 45 [60.0%]) or fewer (7 of 45 [15.6%]), Some studies (18 of 68 [26.47%]) used auxiliary instruments (such as the PTSD symptom scale [61] or the Oswestry Disability Index [70]) as anchors instead, utilising pre-established MICs to define minimum important change on these scales. Notably, some of the rating of change items applied in the literature were symptom-specific [43], with others—especially in the context of cross-sectional studies—requiring participants to provide a point estimate of general or symptom-specific health [89].

## Summary of MIC characteristics

**Fig. 3:**
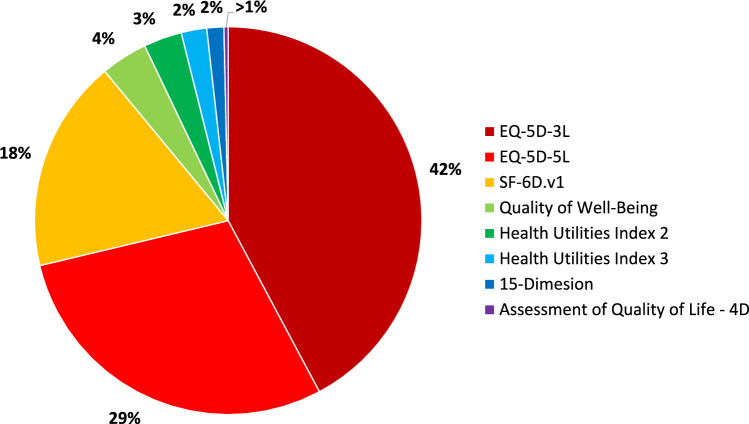
Pie chart representing proportions of minimum importance change estimates to which each instrument relates

Table 4 (supported by Supplementary Table 2) presents a summary of the characteristics of all anchor-based MICs (n = 282). As demonstrated in Supplementary Table 2, only two-thirds of MIC estimates had standard errors [*n* = 184 (65.25%)]. At the MIC level, mean-based approaches to estimation were again the most prevalent (107, 37.94%). This was followed by instrument-defined (60, 21.28%), regression-based (41, 14.54%) and receiver operating characteristic curve (35, 12.41%) methodologies. Table 4 also demonstrates that the most common instruments at the study level were also the most common at the MIC level. Specifically, 42.20% (*n* = 119) of MICs were estimated for the EQ-5D-3L, 29.08% (*n* = 82) for the EQ-5D-5L, and 17.73% (*n* = 50) for the SF-6D.v1 (these data, as well as proportions for other instruments, are graphically represented in Fig. 3). Additionally, we can observe that a plurality of MICs were bi-directional (n = 132, 46.81%), with n = 115 (40.78%) being estimated for improvement specifically.

**Table 4:** Characteristics of the 282 extracted minimum important changes (MICs)

Characteristic	Number	Percentage
Instrument		
EQ-5D-3L	119	42.20
EQ-5D-5L	82	29.08
SF-6D.v1	50	17.73
Quality of well-being	11	3.90
Health Utilities Index 2	9	3.19
Health Utilities Index 3	6	2.13
15-Dimension	4	1.42
Assessment of Quality of Life—4D	1	0.35
Method		
Mean	107	37.94
Difference of means	26	9.22
Median	3	1.06
Percentile	8	2.84
Equipercentile	2	0.71
ROC curve^a^	35	12.41
Regression	41	14.54
Instrument defined	60	21.28
Direction		
Deterioration	35	12.41
Bi-directional	132	46.81
Improvement	115	40.78
Common countries		
Canada	45	15.96
Great Britain	36	12.77
Hong Kong	20	7.09
Japan	15	5.32
Netherlands	19	6.74
Norway	26	9.22
Switzerland	11	3.90
United States	54	19.15

^a^ROC is an abbreviation of Receiver Operating Characteristics

## Summary statistics for MICs

MIC summary statistics are represented in Table 5. They show that the median age of participants in MIC studies was 56.10 years (49.40–65.10 IQR). This indicates that samples from which MICs were obtained were relatively old in age. Conversely, the samples were largely representative of sex distributions, with a 52.30 median percentage of female participants.

**Table 5:** Summary statistics and stratifications for minimum important changes (MICs)

Variable	Summary statistics				
	Mean	Median	25th Perc.	75th Perc.	N
Age^d^	56.61	56.10	49.40	65.1	49
Percentage female^d^	50.89	52.30	41.60	69.20	53
Number of participants^d^	8337	364.00	177.00	1243.00	64
Months to follow-up^d^	9.55	12.00	4.50	12.00	51
Minimum important change	0.071	0.071	0.040	0.106	282
Anchor-instrument correlation	0.45	0.42	0.34	0.55	90
Standard error of measurement	0.12	0.13	0.08	0.13	28
Minimum detectable change	0.40	0.38	0.36	0.49	18
Area under the curve	0.75	0.74	0.69	0.80	32

Variable	Stratifications				
	Mean	Median	25th Perc.	75th Perc.	N
Instrument					
EQ-5D-3L	0.078	0.079	0.040	0.106	74
EQ-5D-5L	0.064	0.069	0.044	0.080	64
SF-6D.v1	0.049	0.040	0.030	0.060	43
Quality of well-being	0.123	0.120	0.030	0.210	7
Health Utilities Index 2	0.037	0.040	0.030	0.045	5
Health Utilities Index 3	0.051	0.051	0.032	0.070	2
15-Dimesion	0.018	0.018	0.010	0.025	4
Method					
Mean	0.071	0.054	0.035	0.100	69
Difference of means	0.076	0.073	0.061	0.093	9
Median	0.037	0.035	0.030	0.047	3
Percentile	0.020	0.015	0.000	0.040	6
ROC curve^b^	0.079	0.075	0.037	0.100	14
Regression	0.043	0.041	0.028	0.052	36
Instrument defined	0.072	0.071	0.049	0.087	60
Direction					
Deterioration	0.064	0.050	0.040	0.070	30
Bi-directional	0.065	0.060	0.035	0.089	107
Improvement	0.069	0.065	0.038	0.090	62
Major medical intervention					
Without intervention	0.066	0.058	0.037	0.089	199
With intervention	0.144	0.113	0.080	0.180	83

^b^ROC is an abbreviation of Receiver Operating Characteristics; ^d^For categories labelled with a *d*, study-level data was provided. Data utilised in the above stratifications was exclusive of MICs associated with major medical interventions, excepting the fourth and final stratification. The Assessment of Quality of Life—4D instrument and the equipercentile method are excluded from this table due to small cell sizes

**Fig. 4:**
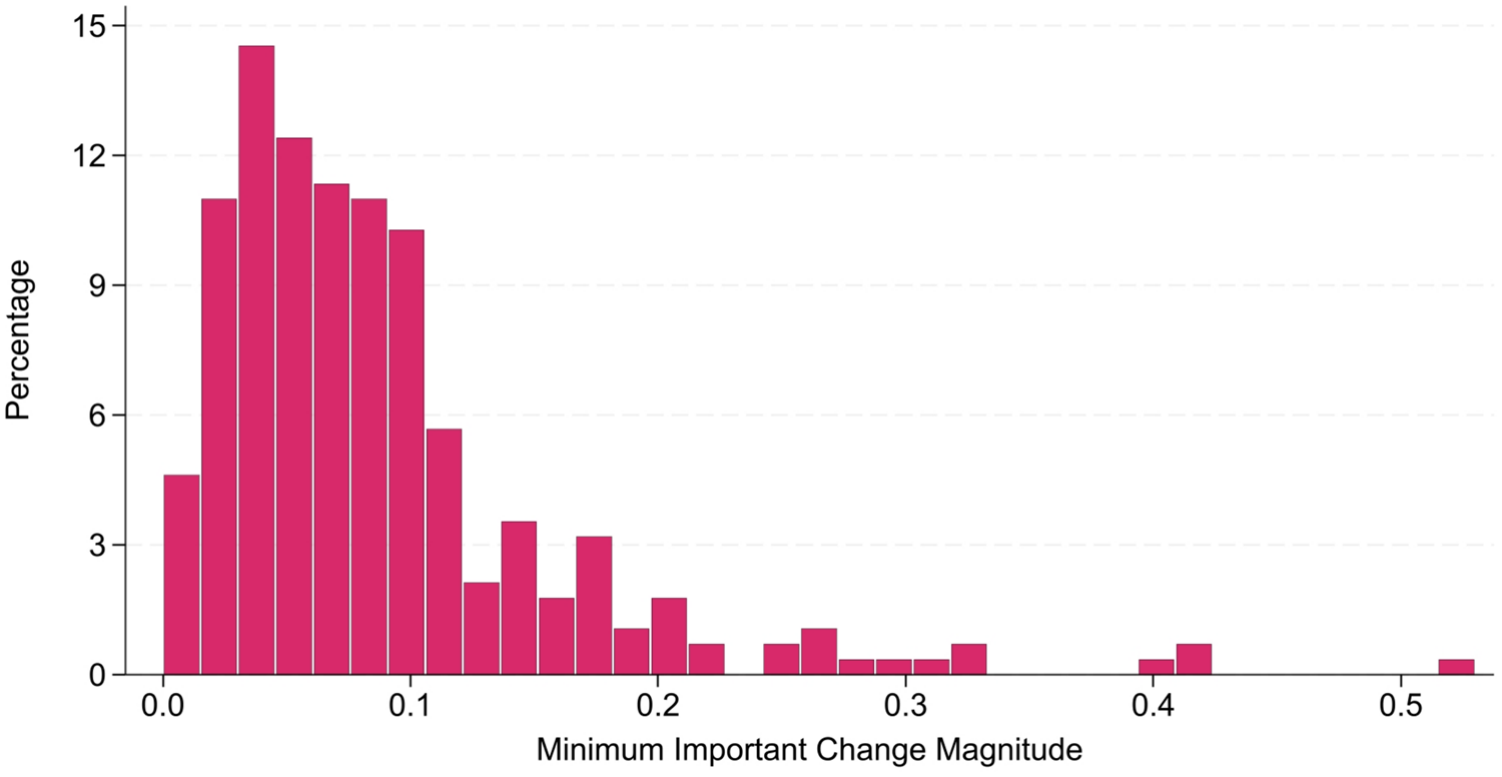
Histogram of minimum importance change MIC values

Figure 4 comprises a histogram of all extracted MIC estimates and shows that most estimates were less than 0.10 in magnitude. The overall mean *and* median MIC was 0.071, the median time to follow-up was 12 months, and the anchor-instrument correlation had a median value of 0.42 (0.34–0.55 IQR), which is well above the 0.243 cut-off for moderate correlation [107]. Interestingly, the median standard error of measurement was in excess of the median MIC value (0.13, 0.08–0.13 IQR). Notably, these standard errors of measurement were sourced for only a small fraction of MICs and may not be representative.

### MIC values stratified by key variables

Table 5 displays mean MIC values stratified over several variables. Importantly, this table indicates that estimation by means (0.071), difference of means (0.076), and the ROC curve (0.079) produced similar results, on average, as did instrument-defined estimation (0.072). Notably, estimation by regression produced substantially smaller MICs (mean of 0.043). Table 5 also shows that the highest mean MIC values were associated with the Quality of Well-Being (QWB) instrument, with the smallest being associated with the 15-Dimension (15-D) instrument. Given the small cell sizes associated with certain instruments, and the heterogeneity present among included studies and MAUIs, instrument-specific variation in MICs should be interpreted with caution.

Stratification also showed that directionality does not impact MIC magnitudes, and that MICs estimated in the context of major medical interventions were—as expected—substantially larger on average (0.144 compared to 0.066). Regarding value-set tariffs, sufficient data was only available for a comparison of EQ-5D-3L MICs estimated under the United States and United Kingdom tariffs; no significant difference was observed [data not shown]. It is notable that in 27.94% (19 of 68) of studies, the value-set tariff used was neither reported nor implicit.

## Discussion

Utilising a comprehensive search strategy, we conducted a systematic review of literature relating to MICs for MAUIs. This review established a concise and comprehensive definition of a MIC (based on extant definitions) and revealed that simple methods of MIC estimation—such as estimation by means and approximation by standard deviation—were most prevalent in the literature. It also demonstrated that MICs vary greatly across instruments and that the choice of estimation methodology can affect MIC estimates profoundly (a finding supported in the general MIC literature [110]). Furthermore, we observed a paucity of MIC estimates for many MAUIs; indeed, we did not identify a single MIC estimated specifically for use with the Assessment of Quality of Life—8D instrument or SF-6D.v2 [111, 112].

Another methodological concern highlighted by our results was that the reporting of key items in the included studies was insufficient, with many studies not establishing concurrent validity (regarding the instrument and anchors) or failing to report MIC estimate precision. Informativeness was not found to be systematically greater in newer publications. Interestingly, poor reporting in the MIC literature generally has been identified previously [113].

Effective estimation and coherent reporting of MICs is of great importance, given that MICs can inform study endpoints and thus influence the validity of study outcomes. As noted by Crosby et al. [122], high-quality MICs are “especially important because small numerical differences in mean HRQOL scores might give statistically significant results when large sample sizes are used, but statistical significance is not equivalent to clinical significance”. In recognition of this—and concordant with this study’s second aim of providing guidance to MIC estimation for MAUIs—recommendations for the estimation of MICs in the context of MAUIs are provided in Table 6, and briefly discussed in the next section.

**Table 6:** Summary of recommendations of minimum important change (MIC) estimation

Category	Recommendations
*Formulating anchors*	Anchor items should comprise seven, nine, or ten levels, with seven being preferable. A three-level sorting item may be employed to increase granularity by directing participants to improvement/deterioration-specific scales [13, 114, 115]
	Consumer and community involvement is essential in formulating anchors, especially regarding face validity, intelligibility, and relevance [116–118]
	A correlation of at least 0.3, ascertained using Spearman’s Rho, between the anchor and instrument is necessary to establish sufficient convergent validity [119]
	A single, global rating of change item (or equivalent) is to be preferred over other instruments and multiple anchors [120]
*Estimation*	MICs should be estimated using parametric modelling or nonparametric receiver operating characteristic curve methods [105]
	The parametric method is to be preferred when the assumptions of logistic regression (including homoscedasticity and a binormal probability distribution function) are met [104, 121]
*Other concerns*	Regression to the mean and ceiling and floor effects must be considered as they can bias MIC estimates [120, 122]
	MICs for improvement and deterioration may differ; pairwise statistical tests can determine if overall or direction-specific MICs are more appropriate [75]
	Recall bias is unavoidable in anchor-based estimation and its potential impact should be tested [119]
	When using the parametric method, variables that may unduly influence MIC estimates should be controlled for in regression [38, 123]
	Due to the subjective nature of patient-reported outcome measures [12, 124], large samples are preferable when estimating MICs

Included references support the recommendations provided

### Recommendations for MIC estimation

Our recommendations relating to MIC estimation were drawn from the literature and often contrasted with the methods applied in the articles included in this review. For example, we noted a frequent usage of distribution-based methods (used in 45.59% of eligible studies), especially the approximation by a half-standard deviation. Notably, the United States Food and Drug Administration indicated that MICs should not be estimated by distribution-based methods alone, writing “Distribution-based methods … do not directly consider the patient voice, and as such, are insufficient to serve as the sole basis for identifying an [MIC]” [100]. Moreover, and more generally, distribution-based measures qua MICs have been indicated to be inappropriate as “*distribution-based indices provide no direct information about*” a MIC [125] or “*guidance regarding the importance of the change*” [109]. Accordingly, our recommendations for MIC estimation pertain to anchor-based methodologies.

To provide guidance regarding the optimal anchor-based method of MIC estimation, we drew from the recommendations provided by Terluin et al*.* [105]. In summary, their study suggested the use of a regression-based predictive modelling approach, which utilised logistic regression and produced similar results to those obtained through estimation by the ROC curve, maximised according to the mathematical rule of Youden’s Index. Interestingly, one of the studies extracted by this review successfully applied the predictive modelling method, demonstrating its applicability [55]. Extending on Terluin et al*.* [105], we also suggested the use of non-parametric ROC curve methods when the assumptions of logistic regression cannot be met.

### Strengths and limitations

Our study was advantaged by an expansive search strategy designed to capture all relevant literature that satisfied our broad inclusion criteria. Its principal strength is that brings together a variety of information, not before summarised and synthesised in one study. Related to this, many of the recommendations for MIC estimation we provided are extensively supported by the literature. This study also benefits from its use of inductive qualitative methods in systematically and concisely describing archetypical methods of MIC estimation. Importantly, we have, to our knowledge, provided the only peer-reviewed guidance for MIC estimation in the context of MAUIs. Furthermore, we have established a reporting checklist and a comprehensive definition of what constitutes a MIC, both of which can be utilised in future studies.

Our study also had some limitations. As meta-analysis was not feasible, our quantitative analyses gave equal weight to each study and MIC, and therefore did not account for study size nor estimate variance. Additionally, in many instances, we sourced multiple MICs from individual papers. To ensure the completeness of our review, only very similar and identical MICs were excluded. MIC-level findings may therefore have been biased by influential studies that made several unique MIC estimates. While we acknowledge that this may have affected our results, the alternative would have been to *exclude* unique MICs from our study, making our systematic review non-comprehensive and incomplete.

The above limitations raise concerns regarding possible bias in our study’s non-narrative, quantitative findings (ROBIS domain four). Importantly, the causes of this bias are intrinsic to the literature that we have reviewed and as such unavoidable. Any systematic review of MICs for MAUIs would encounter similar limitations. As such, we acknowledge that our quantitative syntheses should be interpreted with caution. Contrastingly, the limitations we have described do not extend to our qualitative and narrative findings, nor to the recommendations of our study, which therefore represent categorically unbiased contributions to the literature.

## Conclusions

Our study highlighted some reporting deficiencies in the literature pertaining to MICs for MAUIs. It also demonstrated that, aside from the most common MAUIs (EQ-5D-3L, EQ-5D-5L, and SF-6D.v1), there exists a dearth of MIC estimates. More concerning, some methods frequently employed to estimate MICs for MAUIs did not correspond with what experts have recommended in the literature. In compiling recommendations from the literature, we hope that this study will contribute to improvements in the methods used to estimate MAUI MICs and encourage further research and exposition.

## Supplementary Information

Below is the link to the electronic supplementary material.

Supplementary file1 (DOCX 271 KB)

## Data Availability

Primary data is made available in the supplementary materials.

## Declarations

### Standards of reporting

The PRISMA checklist was adhered to by this study to ensure adequate reporting.
