# Supplementary material for: A systematic review of minimum important changes for generic multi-attribute utility instruments and recommendations for their estimation
Source: Eur J Health Econ. 2025 Apr 16;26(8):1383–99. doi: 10.1007/s10198-025-01778-3 (PMC12572032; doi:10.1007/s10198-025-01778-3)
Supplement: Supplementary file 1 — Supplementary file1 (DOCX 271 KB) [file 10198_2025_1778_MOESM1_ESM.docx]

**Appendix A: Search Methodology**


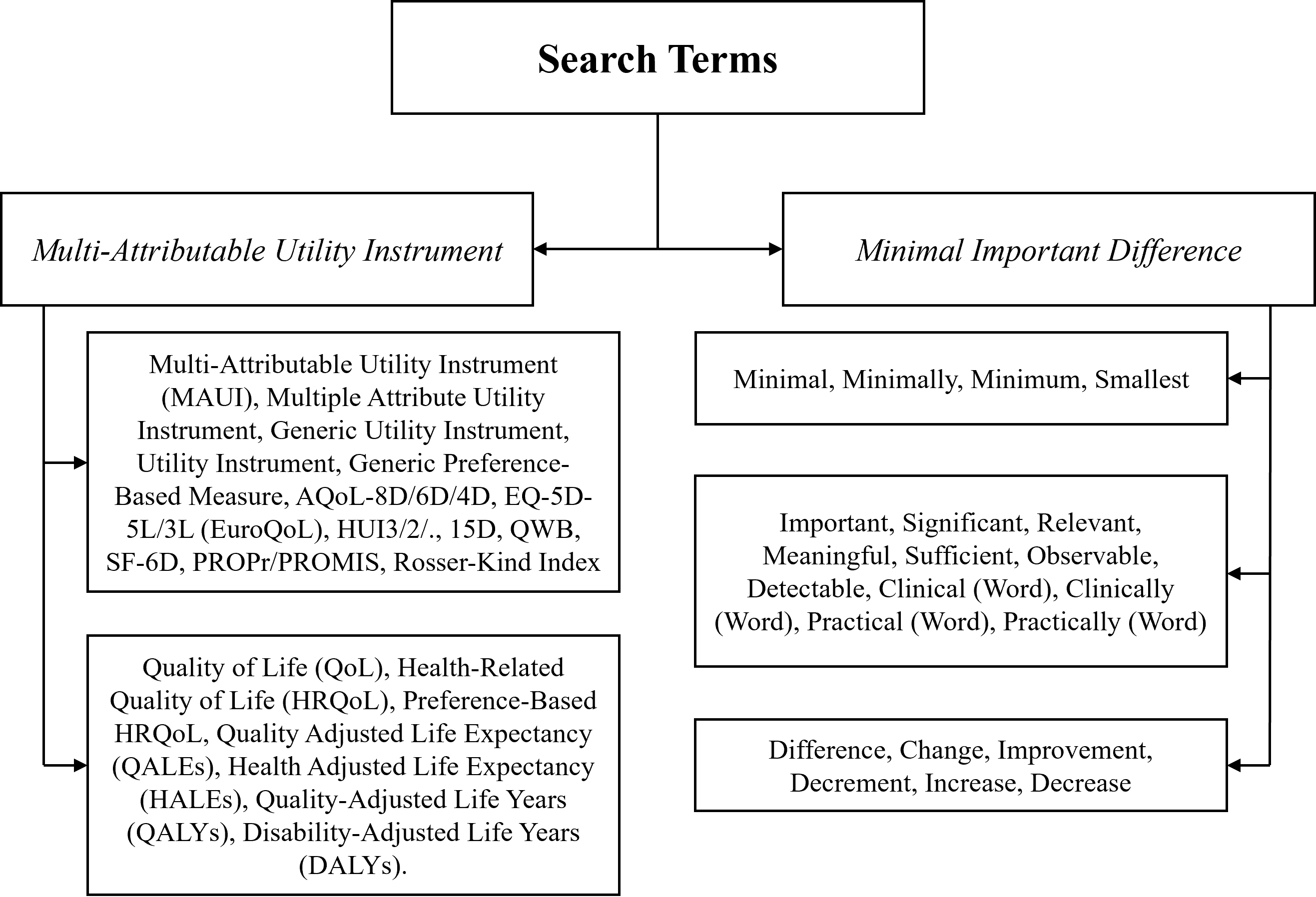


Words associated with ‘minimal important difference’ are divided by which element of the phrase they are interchangeable with. From top to bottom, the words are associated with ‘minimal’, ‘important’, and ‘difference’. Additionally, ‘(Word)’ indicates that singular words from the same category should be added. For example, ‘Clinically’ would become ‘Clinically Important’ and ‘Clinically Significant’. Words associated with ‘Multi-Attribute Utility Instrument’ are divided into (top) instrument names (generic and specific) and (bottom) outcome measures associated with multi-attribute utility instruments.

A pre-study, preliminary search for relevant papers was conducted using the PubMed database. This permitted collection of keywords appropriate for electronic database searches. A professional librarian was enlisted to assist with this task. Collected terms were grouped based on synonymity (see above).

The search strategy selected requires one word or phrase from each of the ‘minimal important difference’ divisions and phrase or name from either ‘multiple attribute utility instrument’ division to be present in an article’s title and/or abstract for that paper to be considered for inclusion. Additionally, search terms will be trialled as pluralised (hyphenated) and singular (non-hyphenated) variants. Relevant acronyms are to be applied in searches, as well as their respective expansions. Note that many phrases synonymous with the technical term (minimal important difference’ are present in the search strategy due to the heterogeneity of their usage and the lack of a firmly established nomenclature ^39^.

Both economic and biomedical electronic databases will be searched in this review, from 01/06/2022 through 07/06/2022. Economic databases to be investigated are the American Economic Association database (EconLit) via EBSCO, the IDEAs database by Research Papers in Economics (RePEc), and the International Health Technology Assessment Database (INAHTA). Biomedical databases that are to be examined include Medline, via PubMed and Ovid; PsycINFO, Embase, Emcare, and the Joanna Briggs Institute Evidence-Based Practice (JBIEBP) database via Ovid; and the Cumulative Index to Nursing and Allied Health Literature (CINAHL), via EBSCO. In addition, we will also search Health Business Elite via EBSCO, and google scholar will be utilised to maximise the review’s completeness.

**Supplementary Table 1:** Study Characteristics

| **No.** | **Title** | **First Author** | **Date of Publication** | **Instruments** | **Methods** | **Data Type** | **Reporting Quality** |
| --- | --- | --- | --- | --- | --- | --- | --- |
| 1 | *Estimating the minimum important change in the 15D scores* | Soili Alanne | 22/08/2014 | 15D | Regression, ROC | Longitudinal | 100.00% |
| 2 | *Defining the minimum clinically important difference for grade I degenerative lumbar spondylolisthesis: insights from the Quality Outcomes Database* | Anthony Asher | 1/01/2018 | EQ-5D-3L | Mean, Difference of Means, ROC, Distribution-Based | Longitudinal | 50.00% |
| 3 | *Validity of EQ-5D utility index and minimal clinically important difference estimation among patients with chronic obstructive pulmonary disease* | Eunmi Bae | 23/03/2020 | EQ-5D-3L | Regression, Distribution-Based | Cross-Sectional | 75.00% |
| 4 | *Psychometric properties of the EQ-5D-5L in patients with hip or knee osteoarthritis: reliability, validity and responsiveness* | Amaia Bilbao | 5/07/2018 | EQ-5D-3L | Mean | Longitudinal | 25.00% |
| 5 | *Mathematical coupling may account for the association between baseline severity and minimally important difference values* | John Browne | 21/10/2009 | EQ-5D-3L | Difference of Means | Longitudinal | 75.00% |
| 6 | *Validity, responsiveness, and minimal clinically important difference of EQ-5D-5L in stroke patients undergoing rehabilitation* | Poyu Chen | 30/11/2015 | EQ-5D-5L | Mean, Distribution-Based | Longitudinal | 50.00% |
| 7 | *Comparing the validity and responsiveness of the EQ-5D-5L to the Oxford hip and knee scores and SF-12 in osteoarthritis patients 1 year following total joint replacement* | Barbara Conner-Spady | 8/02/2018 | EQ-5D-5L | Mean | Longitudinal | 50.00% |
| 8 | *Effects of vedolizumab on health-related quality of life in patients with ulcerative colitis: results from the randomised GEMINI 1 trial* | Brian Feagan | 17/11/2016 | EQ-5D-3L | Distribution Based | RCT | NA |
| 9 | *SF-6D utility index as measure of minimally important difference in health status change* | Pranav Gandhi | 1/01/2012 | SF-6D.v1 | Median, Distribution-Based | RCT | 75.00% |
| 10 | *A Cost-Effectiveness Comparison Between Open Transforaminal and Minimally Invasive Lateral Lumbar Interbody Fusions Using the Incremental Cost-Effectiveness Ratio at 2-Year Follow-up* | Gurpreet Gandhoke | 30/12/2015 | EQ-5D-3L | ROC | Longitudinal | 50.00% |
| 11 | *The Responsiveness of Quality of Life Utilities to Change in Depression: A Comparison of Instruments (SF-6D, EQ-5D, and DFD)* | Sylvia Gerhards | 6/05/2011 | EQ-5D-3L and SF-6D.v1 | Mean | RCT | 75.00% |
| 12 | *Use of large-scale HRQoL datasets to generate individualised predictions ad inform patients about the likely benefit of surgery* | Nils Gutacker | 31/05/2017 | EQ-5D-3L | Difference of Means | Longitudinal | 50.00% |
| 13 | *Responsiveness and Minimally Important Difference of SF-6D and EQ-5D Utility Scores for the Treatment of Pelvic Organ Prolapse* | Heidi Harvie | 22/11/2018 | EQ-5D-3L  and SF-6D.v1 | Difference of Means, Regression, Distribution-Based | RCT | 50.00% |
| 14 | *Population norms and meaningful differences for the Assessment of Quality of Life (AQoL) measure.* | Graeme Hawthorne | 1/10/2004 | AQoL-4D | Mean | Longitudinal | 50.00% |
| 15 | *Estimation of an Instrument-Defined Minimally Important Difference in EQ-5D-5L Index Scores Based on Scoring Algorithms Derived Using the EQ-VT Version 2 Valuation Protocols* | Edward Henry | 21/07/2020 | EQ-5D-5L | Instrument-Defined | Value-Set | NA |
| 16 | *Responsiveness and minimal clinically important difference for the EQ-5D in chronic rhinosinusitis* | Lloyd Hoehle | 1/04/2019 | EQ-5D-3L | Difference of Means, ROC, Distribution-Based | Longitudinal | 100.00% |
| 17 | *Nephrectomy Complication is a Risk Factor of Clinically Meaningful Decrease in Health Utility Among Living Kidney Donors* | Kossar Hosseini | 29/07/2017 | EQ-5D-3L  and SF-6D.v1 | Mean, Distribution-Based | Longitudinal | 75.00% |
| 18 | *The early outcome of surgical treatment for femoroacetabular impingement: success depends on how you measure it* | Franco Impellizzeri | 30/03/2012 | EQ-5D-3L | ROC | Longitudinal | 50.00% |
| 19 | *Minimally important differences and predictors of change in quality of life in type 2 diabetes: A community-based survey in China* | Xuejing Jin | 31/08/2018 | EQ-5D-3L | Mean, ROC, Distribution-Based | Longitudinal | 0.00% |
| 20 | *Comparison of the SF6D, the EQ5D, and the oswestry disability index in patients with chronic low back pain and degenerative disk disease* | Lars Johnsen | 26/04/2013 | EQ-5D-3L  and SF-6D.v1 | ROC | RCT | 75.00% |
| 21 | *The Minimally Clinically Important Difference in Generic Utility-Based Measures* | Robert Kaplan | 1/03/2005 | QWB-SA | Distribution-Based | RCT | NA |
| 22 | *Minimum clinically important difference in outcome scores among patients undergoing cervical laminoplasty* | So Kato | 14/03/2019 | EQ-5D-3L | ROC | Longitudinal | 50.00% |
| 23 | *Reliability, validity, and minimally important differences of the SF-6D in systemic sclerosis* | Dinesh Khanna | 3/04/2007 | SF-6D.v1 | Mean | Cross-Sectional and RCT | 100.00% |
| 24 | *Estimation of minimally important differences in the EQ-5D and SF-6D indices and their utility in stroke* | Sang-Kyu Kim | 9/03/2015 | EQ-5D-3L  and SF-6D.v1 | Mean | Longitudinal | 75.00% |
| 25 | *Estimating a minimal clinically important difference for the EuroQol 5-dimension health status index in persons with multiple sclerosis* | Christine Kohn | 5/05/2014 | EQ-5D-3L | Distribution-Based | Cross-Sectional | NA |
|  |  |  |  |  |  |  |  |
| 26 | *Responsiveness and minimal important score differences in quality-of-life questionnaires: a comparison of the EORTC QLQ-C30 cancer specific questionnaire to the generic utility questionnaires EQ-5D and 15D in patients with multiple myeloma* | Ann Kvam | 26/07/2011 | EQ-5D-3L  and 15D | Mean, Distribution-Based | Longitudinal | 25.00% |
| 27 | *Identification of Cutpoints for Acceptable Health Status and Important Improvement in Patient-Reported Outcomes, in Rheumatoid Arthritis, Psoriatic Arthritis and Ankylosing Spondylitis* | Maria Kvamme | 1/12/2009 | EQ-5D-3L  and SF-6D.v1 | Percentile, ROC | Longitudinal | 75.00% |
| 28 | *A comparison of the measurement properties and estimation of minimal important differences of the EQ-5D and SF-6D utility measures in patients with systemic sclerosis* | Linda Kwakkenbos | 22/07/2013 | EQ-5D-3L  and SF-6D.v1 | Mean | Longitudinal | 75.00% |
| 29 | *Validity and responsiveness of the EQ-5D-5L and SF-6D in patients with health complaints attributed to their amalgam fillings: a prospective cohort study of patients undergoing amalgam removal* | Admassu Lamu | 17/04/2021 | EQ-5D-3L  and SF-6D.v1 | Mean, Regression, Distribution-Based | Longitudinal | 75.00% |
| 30 | *Minimal clinically important differences for the EQ-5D and the QWB-SA in Post-traumatic Stress Disorder (PTSD): results from a Doubly Randomised Preference Trial (DRPT)* | Quang Le | 12/04/2013 | EQ-5D-3L  and QWB-SA | Regression, ROC, Distribution-Based | RCT | 100.00% |
| 31 | *Validity, responsiveness, and minimal important difference for the SF-6D health utility scale in a spinal cord injured population* | Bonsan Lee | 11/01/2008 | SF-6D.v1 | Mean | RCT | 50.00% |
| 32 | *Measurement and Variation in Estimation of Quality of Life Effects of Patients Undergoing Treatment for Papillary Thyroid Carcinoma* | Carrie Lubitz | 15/12/2016 | EQ-5D-5L | Distribution-Based | Longitudinal | NA |
| 33 | *Using Instrument-Defined Health State Transitions to Estimate Minimally Important Differences for Four Preference-Based Health-Related Quality of Life Instruments* | Nan Luo | 1/04/2010 | EQ-5D-3L,  HUI2, HUI3, and SF-6D.v1 | Instrument-Defined | Value-Set | NA |
| 34 | *Validity, responsiveness and minimal important change of the EQ-5D-5L in patients after rotator cuff repair, shoulder arthroplasty or thumb carpometacarpal arthroplasty* | Miriam Marks | 10/05/2021 | EQ-5D-5L | Mean, Difference of Means, ROC, Distribution-Based | Longitudinal | 50.00% |
| 35 | *A comparison of generic, indirect utility measures (the HUI2, HUI3, SF-6D, and the EQ-5D) and disease-specific instruments (the RAQoL and the HAQ) in rheumatoid arthritis* | Carlo Marra | 18/12/2004 | EQ-5D-3L,  HUI2, HUI3, and SF-6D.v1 | Regression, Distribution-Based | Cross-Sectional | 75.00% |
| 36 | *Measurement properties of the EuroQoL EQ-5D-5L to assess quality of life in patients undergoing carpal tunnel release* | C Marti | 20/07/2016 | EQ-5D-5L | Mean | Longitudinal | 50.00% |
| 37 | *Instrument Defined Estimates of the Minimally Important Difference for the EQ-5D-5L Index Scores* | Nathan McClure | 10/01/2017 | EQ-5D-5L | Instrument-Defined | Value-Set | NA |
| 38 | *Minimally Important Difference of the EQ-5D-5L Index Score in Adults with Type 2 Diabetes* | Nathan McClure | 4/04/2018 | EQ-5D-5L | Instrument-Defined | Cross-Sectional and Value-Set | 50.00% |
| 39 | *A Longitudinal Comparison of Five Preference-weighted Health State Classification Systems in Persons with Intervertebral Disc Herniation* | Christine McDonough | 22/11/2010 | EQ-5D-3L, HUI2, HUI3, SF-6D.v1, and eQWB | Mean | Longitudinal | 50.00% |
| 40 | *Comparing 15D Valuation Studies in Norway and Finland - Challenges When Combining Information from Several Valuation Tasks* | Yvonne Michel | 8/11/2017 | 15D | Instrument-Defined | Value-Set | NA |
| 41 | *Defining Clinically Meaningful Thresholds for Patient-Reported Outcomes in Knee Arthroplasty* | Jasper Most | 5/02/2022 | EQ-5D-3L | Regression, ROC | Longitudinal | 100.00% |
| 42 | *Investigating the minimally important difference of the Diabetes Health Profile (DHP-18) and the EQ-5D and SF-6D in a UK diabetes mellitus population* | Brendan Mulhern | 6/06/2013 | EQ-5D-3L and SF-6D.v1 | Difference of Means, Distribution-Based | Longitudinal | 50.00% |
| 43 | *Minimal clinically important difference in patients who underwent decompression alone for lumbar degenerative disease* | Hiroyuki Nakarai | 23/10/2021 | EQ-5D-3L | Mean, ROC | Longitudinal | 75.00% |
| 44 | *Separating gains and losses in health when calculating the minimum important difference for mapped utility measures* | Michael Nichol | 10/07/2008 | SF-6D.v1 and HUI2 | Mean, Distribution-Based | Longitudinal | 50.00% |
| 45 | *The EQ-5D-5: health status questionnaire in COPD: validity, responsiveness and minimum important difference* | Claire Nolan | 30/03/2016 | EQ-5D-5L | Regression-ROC, Distribution-Based | Longitudinal | 75.00% |
| 46 | *ISSLS prize in clinical science 2020: the reliability and interpretability of score change in lumbar spine research.* | Catharina Parai | 23/11/2019 | EQ-5D-3L | ROC | Longitudinal | 50.00% |
| 47 | *Minimal clinically important improvement (MCII) and patient acceptable symptom state (PASS) in total hip arthroplasty (THA) patients 1 year postoperatively* | Aksel Paulsen | 29/11/2013 | EQ-5D-3L | Mean, Percentile, ROC | Longitudinal | 100.00% |
| 48 | *Applied Machine Learning for Spine Surgeons: Predicting Outcome for Patients Undergoing Treatment for Lumbar Disc Herniation Using PRO Data* | Casper Pedersen | 18/11/2020 | EQ-5D-3L | ROC | Longitudinal | 75.00% |
| 49 | *Estimation of minimally important differences in EQ-5D utility and VAS scores in cancer* | A. Simon Pickard | 21/12/2007 | EQ-5D-3L | Mean, Distribution-Based | Cross-Sectional | 25.00% |
| 50 | *Quality of life assessed with EQ-5D in patients undergoing glioma surgery: What is the responsiveness and minimal clinically important difference?* | Lisa Sagberg | 7/12/2013 | EQ-5D-3L | Mean, Distribution-Based | Cross-Sectional and Longitudinal | 50.00% |
| 51 | *Correlation of the Patient Reported Outcomes Measurement Information System with Legacy Outcomes Measures in Assessment of Response to Lumbar Transforaminal Epidural Steroid Injections* | Leili Shahgholi | 22/01/2015 | EQ-5D-3L | Distribution-Based | Longitudinal | NA |
| 52 | *Long-term patient reported outcomes from a phase III randomized prospective trial of conventional versus hypofractionated radiotherapy for localized prostate cancer* | Talha Shaikh | 28/12/2016 | EQ-5D-3L | Distribution -Based | RCT | NA |
| 53 | *The validity and responsiveness of three quality of life measures in the assessment of psoriasis patients: results of a phase II study* | Richard Shikiar | 27/09/2006 | EQ-5D-3L | Difference of Means, Distribution-Based | RCT | 75.00% |
| 54 | *Japanese population norms for preference-based measures: EQ-5D-3L, EQ-5D-5L, and SF-6D* | Takeru Shiroirwa | 25/08/2015 | EQ-5D-3L,  EQ-5D-5L  and SF-6D.v1 | Difference of Means | Cross-Sectional | 0.00% |
| 55 | *Responsiveness of outcome measures in patients with superior labral anterior and posterior lesions* | Øystein Skare | 27/05/2014 | EQ-5D-3L | ROC | Longitudinal | 50.00% |
| 56 | *Validity, Reliability, and Responsiveness of the EQ-5D in Inflammatory Bowel Disease in Germany* | Renee Stark | 27/05/2009 | EQ-5D-3L | Regression | Longitudinal | 25.00% |
| 57 | *Assessment of the minimum clinically important difference in quality of life in schizophrenia measured by the Quality of Well-Being Scale and disease-specific measures* | Soe Soe Thwin | 7/03/2013 | QWB-SA | Mean, Equipercentile | RCT | 25.00% |
| 58 | *Minimum important difference of the EQ-­5D-­5L and EQ-­VAS in fibrotic interstitial lung disease* | Amy Tsai | 6/10/2020 | EQ-5D-5L | Regress, Distribution-Based | Cross-Sectional | 50.00% |
| 59 | *EQ-5D-derived health utilities and minimally important differences for chronic health conditions: 2011 Commonwealth Fund Survey of Sicker Adults in Canada* | Kate Tsiplova | 15/06/2015 | EQ-5D-3L | Regress, Distribution-Based | Cross-Sectional | 50.00% |
| 60 | *Minimal Clinically Important Change for Pain Intensity, Functional Status, and General Health Status in Patients With Nonspecific Low Back Pain* | Nicole van der Roer | 1/03/2006 | EQ-5D-3L | Mean, ROC, Distribution-Based | RCT | 75.00% |
| 61 | *Defining a Minimum Clinically Important Difference in Patient-Reported Outcome Measures in Lumbar Tubular Microdecompression Patients* | Trent Vanhorn | 31/07/2020 | EQ-5D-5L | ROC | Longitudinal | 25.00% |
| 62 | *What is the relationship between the minimally important difference and health state utility values? The case of the SF-6D* | Stephen Walters | 11/04/2003 | SF-6D.v1 | Mean, Distribution-Based | Longitudinal | 75.00% |
| 63 | *Comparison of the minimally important difference for two health state utility measures: EQ-5D and SF-6D* | Stephen Walters | 1/08/2005 | EQ-5D-3L  and SF-6D.v1 | Mean, Distribution-Based | Longitudinal and RCT | 75.00% |
| 64 | *Measuring the impact of chronic conditions and associated multimorbidity on health related quality of life in the general population in Hong Kong SAR, China: A cross-sectional study* | Eliza Wong | 20/11/2019 | EQ-5D-5L | Instrument-Defined | Cross-Sectional and Value-Set | NA |
| 65 | *Health‑related quality of life in elderly people with hypertension and the estimation of minimally important difference using EQ‑5D‑5L in Hong Kong SAR, China* | Eliza Wong | 2/04/2020 | EQ-5D-5L | Instrument-Defined | Cross-Sectional and Value-Set | NA |
| 66 | *Estimation of minimally important difference of the EQ-­5D-­5L utility scores among patients with either hypertension or diabetes or both: a cross-­sectional study in Hong Kong* | Richard Xu | 26/11/2020 | EQ-5D-5L | Instrument-Defined | Cross-Sectional and Value-Set | NA |
| 67 | *Health-Related Quality of Life of People with Self-Reported Hypertension: A National Cross-Sectional Survey in China* | Qiang Yao | 16/05/2019 | EQ-5D-3L | Distribution-Based | Cross-Sectional | NA |
| 68 | *Estimation of minimal clinically important change of the Japanese version of EQ-5D in patients with chronic noncancer pain: a retrospective research using real-world data* | Kazetake Yoshizawa | 1/03/2016 | EQ-5D-3L | ROC | Longitudinal | 50.00% |

**Supplementary Table 2:** Minimum Important Change (MIC) Characteristics

Legend: LCI (Lower Confidence Interval), UCI (Upper Confidence Interval), Corr. (Correlation between anchor and instrument), SbMD (Anchor measured a Small but Meaningful Difference), n (Number of study participants), Maj. Int. (MIC estimated in the context of a Major Medical Intervention).

| **No.** | **Title** | **First Author** | **MIC** | **LCI** | **UCI** | **Corr.** | **SbMD** | **n** | **Instrument** | **Method** | **Disease** | **Maj. Int.** | **Country** |
| --- | --- | --- | --- | --- | --- | --- | --- | --- | --- | --- | --- | --- | --- |
| 1 | Estimating the minimum important change in the 15D scores | Soili Alanne | 0.015 |  |  | 0.347 | 1 | 4950 | 15D | ROC Curve |  | NO | Finland |
| 2 | Validity of EQ-5D utility index and minimal clinically important difference estimation among patients with chronic obstructive pulmonary disease | Eunmi Bae | 0.028 | 0.023 | 0.034 | 0.551 | 1 | 298 | EQ-5D-3L | Regression | Chronic Obstructive Pulmonary Disorder | NO | South Korea |
| 3 | Psychometric properties of the EQ-5D-5L in patients with hip or knee osteoarthritis: reliability, validity and responsiveness | Amaia Bilbao | 0.07 |  |  |  | 1 | 514 | EQ-5D-5L | Mean | Knee and Hip Osteoarthritis | NO | Spain |
| 4 | Psychometric properties of the EQ-5D-5L in patients with hip or knee osteoarthritis: reliability, validity and responsiveness | Amaia Bilbao | 0.05 |  |  |  | 1 | 514 | EQ-5D-5L | Mean | Knee and Hip Osteoarthritis | NO | Spain |
| 5 | Psychometric properties of the EQ-5D-5L in patients with hip or knee osteoarthritis: reliability, validity and responsiveness | Amaia Bilbao | 0.32 |  |  |  | 1 | 130 | EQ-5D-5L | Mean | Knee and Hip Osteoarthritis | YES | Spain |
| 6 | Mathematical coupling may account for the association between baseline severity and minimally important difference values | John Browne | 0.03 |  |  | 0.24 | 1 | 441 | EQ-5D-3L | Difference of Means | Hernia | YES | Great Britain |
| 7 | Mathematical coupling may account for the association between baseline severity and minimally important difference values | John Browne | 0.04 |  |  | 0.33 | 1 | 269 | EQ-5D-3L | Difference of Means | Varicose Veins | YES | Great Britain |
| 8 | Mathematical coupling may account for the association between baseline severity and minimally important difference values | John Browne | 0.13 |  |  | 0.22 | 1 | 445 | EQ-5D-3L | Difference of Means | Hip Arthroplasty | YES | Great Britain |
| 9 | Mathematical coupling may account for the association between baseline severity and minimally important difference values | John Browne | 0.15 |  |  | 0.42 | 1 | 461 | EQ-5D-3L | Difference of Means | Knee Arthroplasty | YES | Great Britain |
| 10 | Validity, responsiveness, and minimal clinically important difference of EQ-5D-5L in stroke patients undergoing rehabilitation | Poyu Chen | 0.10 |  |  |  | 1 | 65 | EQ-5D-5L | Mean | Stroke | NO | Republic of China |
| 11 | Comparing the validity and responsiveness of the EQ-5D-5L to the Oxford hip and knee scores and SF-12 in osteoarthritis patients 1 year following total joint replacement | Barbara Conner-Spady | 0.22 |  |  | 0.72 | 1 | 269 | EQ-5D-5L | Mean | Osteoarthritis | YES | Canada |
| 12 | Comparing the validity and responsiveness of the EQ-5D-5L to the Oxford hip and knee scores and SF-12 in osteoarthritis patients 1 year following total joint replacement | Barbara Conner-Spady | 0.41 |  |  | 0.72 | 1 | 269 | EQ-5D-5L | Mean | Osteoarthritis | YES | Canada |
| 13 | Comparing the validity and responsiveness of the EQ-5D-5L to the Oxford hip and knee scores and SF-12 in osteoarthritis patients 1 year following total joint replacement | Barbara Conner-Spady | 0.30 |  |  | 0.72 | 1 | 269 | EQ-5D-5L | Mean | Osteoarthritis | YES | Canada |
| 14 | Comparing the validity and responsiveness of the EQ-5D-5L to the Oxford hip and knee scores and SF-12 in osteoarthritis patients 1 year following total joint replacement | Barbara Conner-Spady | 0.20 |  |  | 0.74 | 1 | 268 | EQ-5D-5L | Mean | Osteoarthritis | YES | Canada |
| 15 | Comparing the validity and responsiveness of the EQ-5D-5L to the Oxford hip and knee scores and SF-12 in osteoarthritis patients 1 year following total joint replacement | Barbara Conner-Spady | 0.28 |  |  | 0.74 | 1 | 268 | EQ-5D-5L | Mean | Osteoarthritis | YES | Canada |
| 16 | Comparing the validity and responsiveness of the EQ-5D-5L to the Oxford hip and knee scores and SF-12 in osteoarthritis patients 1 year following total joint replacement | Barbara Conner-Spady | 0.26 |  |  | 0.74 | 1 | 268 | EQ-5D-5L | Mean | Osteoarthritis | YES | Canada |
| 17 | SF-6D utility index as measure of minimally important difference in health status change | Pranav Gandhi | 0.035 | -0.155 | 0.225 | 0.15 | 1 | 894 | SF-6D.v1 | Mean | Hypertension and Coronary Artery Disease | NO | United States |
| 18 | SF-6D utility index as measure of minimally important difference in health status change | Pranav Gandhi | 0.030 | -0.158 | 0.218 | 0.15 | 1 | 894 | SF-6D.v1 | Mean | Hypertension and Coronary Artery Disease | NO | United States |
| 19 | SF-6D utility index as measure of minimally important difference in health status change | Pranav Gandhi | 0.047 | -0.145 | 0.239 | 0.15 | 1 | 894 | SF-6D.v1 | Mean | Hypertension and Coronary Artery Disease | NO | United States |
| 20 | A Cost-Effectiveness Comparison Between Open Transforaminal and Minimally Invasive Lateral Lumbar Interbody Fusions Using the Incremental Cost-Effectiveness Ratio at 2-Year Follow-up | Gurpreet Gandhoke | 0.25 |  |  |  | 1 | 44 | EQ-5D-3L | ROC Curve | Degenerative Spondylosis | YES | United States |
| 21 | A Cost-Effectiveness Comparison Between Open Transforaminal and Minimally Invasive Lateral Lumbar Interbody Fusions Using the Incremental Cost-Effectiveness Ratio at 2-Year Follow-up | Gurpreet Gandhoke | 0.26 |  |  |  | 1 | 29 | EQ-5D-3L | ROC Curve | Degenerative Spondylosis | YES | United States |
| 22 | The Responsiveness of Quality of Life Utilities to Change in Depression: A Comparison of Instruments (SF-6D, EQ-5D, and DFD) | Sylvia Gerhards | 0.03 | -0.016 | 0.076 | 0.379 | 1 | 267 | EQ-5D-3L | Mean | Depression | NO | Netherlands |
| 23 | The Responsiveness of Quality of Life Utilities to Change in Depression: A Comparison of Instruments (SF-6D, EQ-5D, and DFD) | Sylvia Gerhards | 0.04 | 0.008 | 0.072 | 0.379 | 1 | 267 | EQ-5D-3L | Mean | Depression | NO | Netherlands |
| 24 | The Responsiveness of Quality of Life Utilities to Change in Depression: A Comparison of Instruments (SF-6D, EQ-5D, and DFD) | Sylvia Gerhards | 0.01 | -0.15 | 0.17 | 0.437 | 1 | 267 | SF-6D.v1 | Mean | Depression | NO | Netherlands |
| 25 | The Responsiveness of Quality of Life Utilities to Change in Depression: A Comparison of Instruments (SF-6D, EQ-5D, and DFD) | Sylvia Gerhards | 0.03 | -0.13 | 0.19 | 0.437 | 1 | 267 | SF-6D.v1 | Mean | Depression | NO | Netherlands |
| 26 | Use of large-scale HRQoL datasets to generate individualised predictions ad inform patients about the likely benefit of surgery | Nils Gutacker | 0.106 | 0.095 | 0.116 |  | 1 | 185111 | EQ-5D-3L | Difference of Means |  | YES | Great Britain |
| 27 | Use of large-scale HRQoL datasets to generate individualised predictions ad inform patients about the likely benefit of surgery | Nils Gutacker | 0.091 | 0.075 | 0.106 |  | 1 | 185111 | EQ-5D-3L | Difference of Means |  | YES | Great Britain |
| 28 | Use of large-scale HRQoL datasets to generate individualised predictions ad inform patients about the likely benefit of surgery | Nils Gutacker | 0.090 | 0.083 | 0.097 |  | 1 | 198007 | EQ-5D-3L | Difference of Means |  | YES | Great Britain |
| 29 | Use of large-scale HRQoL datasets to generate individualised predictions ad inform patients about the likely benefit of surgery | Nils Gutacker | 0.081 | 0.071 | 0.090 |  | 1 | 198007 | EQ-5D-3L | Difference of Means |  | YES | Great Britain |
| 30 | Use of large-scale HRQoL datasets to generate individualised predictions ad inform patients about the likely benefit of surgery | Nils Gutacker | 0.041 | 0.033 | 0.048 |  | 1 | 114605 | EQ-5D-3L | Difference of Means |  | YES | Great Britain |
| 31 | Use of large-scale HRQoL datasets to generate individualised predictions ad inform patients about the likely benefit of surgery | Nils Gutacker | 0.069 | 0.056 | 0.081 |  | 1 | 114605 | EQ-5D-3L | Difference of Means |  | YES | Great Britain |
| 32 | Responsiveness and Minimally Important Difference of SF-6D and EQ-5D Utility Scores for the Treatment of Pelvic Organ Prolapse | Heidi Harvie | 0.025 | -0.005 | 0.055 |  | 1 | 715 | EQ-5D-3L | Regression | Pelvic Organ Prolapse | YES | United States |
| 33 | Responsiveness and Minimally Important Difference of SF-6D and EQ-5D Utility Scores for the Treatment of Pelvic Organ Prolapse | Heidi Harvie | 0.026 | 0.007 | 0.045 |  | 1 | 1100 | SF-6D.v1 | Regression | Pelvic Organ Prolapse | YES | United States |
| 34 | Population norms and meaningful differences for the Assessment of Quality of Life (AQoL) measure. | Graeme Hawthorne | 0.060 | 0.030 | 0.080 |  | 1 | 3863 | AQoL-4D | Difference of Means |  | YES | Australia |
| 35 | Estimation of an Instrument-Defined Minimally Important Difference in EQ-5D-5L Index Scores Based on Scoring Algorithms Derived Using the EQ-VT Version 2 Valuation Protocols | Edward Henry | 0.083 | 0.081 | 0.085 |  | 1 |  | EQ-5D-5L | Instrument Defined |  | NO | Germany |
| 36 | Estimation of an Instrument-Defined Minimally Important Difference in EQ-5D-5L Index Scores Based on Scoring Algorithms Derived Using the EQ-VT Version 2 Valuation Protocols | Edward Henry | 0.093 | 0.091 | 0.097 |  | 1 |  | EQ-5D-5L | Instrument Defined |  | NO | Indonesia |
| 37 | Estimation of an Instrument-Defined Minimally Important Difference in EQ-5D-5L Index Scores Based on Scoring Algorithms Derived Using the EQ-VT Version 2 Valuation Protocols | Edward Henry | 0.098 | 0.096 | 0.101 |  | 1 |  | EQ-5D-5L | Instrument Defined |  | NO | Ireland |
| 38 | Estimation of an Instrument-Defined Minimally Important Difference in EQ-5D-5L Index Scores Based on Scoring Algorithms Derived Using the EQ-VT Version 2 Valuation Protocols | Edward Henry | 0.072 | 0.072 | 0.072 |  | 1 |  | EQ-5D-5L | Instrument Defined |  | NO | Malaysia |
| 39 | Estimation of an Instrument-Defined Minimally Important Difference in EQ-5D-5L Index Scores Based on Scoring Algorithms Derived Using the EQ-VT Version 2 Valuation Protocols | Edward Henry | 0.080 | 0.077 | 0.083 |  | 1 |  | EQ-5D-5L | Instrument Defined |  | NO | Poland |
| 40 | Estimation of an Instrument-Defined Minimally Important Difference in EQ-5D-5L Index Scores Based on Scoring Algorithms Derived Using the EQ-VT Version 2 Valuation Protocols | Edward Henry | 0.080 | 0.079 | 0.082 |  | 1 |  | EQ-5D-5L | Instrument Defined |  | NO | Portugal |
| 41 | Estimation of an Instrument-Defined Minimally Important Difference in EQ-5D-5L Index Scores Based on Scoring Algorithms Derived Using the EQ-VT Version 2 Valuation Protocols | Edward Henry | 0.101 | 0.098 | 0.104 |  | 1 |  | EQ-5D-5L | Instrument Defined |  | NO | Republic of China |
| 42 | Estimation of an Instrument-Defined Minimally Important Difference in EQ-5D-5L Index Scores Based on Scoring Algorithms Derived Using the EQ-VT Version 2 Valuation Protocols | Edward Henry | 0.078 | 0.076 | 0.084 |  | 1 |  | EQ-5D-5L | Instrument Defined |  | NO | United States |
| 43 | Estimation of an Instrument-Defined Minimally Important Difference in EQ-5D-5L Index Scores Based on Scoring Algorithms Derived Using the EQ-VT Version 2 Valuation Protocols | Edward Henry | 0.152 | 0.120 | 0.184 |  | 1 |  | EQ-5D-3L | Instrument Defined |  | NO | Denmark |
| 44 | Estimation of an Instrument-Defined Minimally Important Difference in EQ-5D-5L Index Scores Based on Scoring Algorithms Derived Using the EQ-VT Version 2 Valuation Protocols | Edward Henry | 0.117 | 0.081 | 0.153 |  | 1 |  | EQ-5D-3L | Instrument Defined |  | NO | Germany |
| 45 | Estimation of an Instrument-Defined Minimally Important Difference in EQ-5D-5L Index Scores Based on Scoring Algorithms Derived Using the EQ-VT Version 2 Valuation Protocols | Edward Henry | 0.097 | 0.076 | 0.118 |  | 1 |  | EQ-5D-3L | Instrument Defined |  | NO | Japan |
| 46 | Estimation of an Instrument-Defined Minimally Important Difference in EQ-5D-5L Index Scores Based on Scoring Algorithms Derived Using the EQ-VT Version 2 Valuation Protocols | Edward Henry | 0.124 | 0.098 | 0.150 |  | 1 |  | EQ-5D-3L | Instrument Defined |  | NO | Netherlands |
| 47 | Estimation of an Instrument-Defined Minimally Important Difference in EQ-5D-5L Index Scores Based on Scoring Algorithms Derived Using the EQ-VT Version 2 Valuation Protocols | Edward Henry | 0.160 | 0.129 | 0.191 |  | 1 |  | EQ-5D-3L | Instrument Defined |  | NO | Spain |
| 48 | Estimation of an Instrument-Defined Minimally Important Difference in EQ-5D-5L Index Scores Based on Scoring Algorithms Derived Using the EQ-VT Version 2 Valuation Protocols | Edward Henry | 0.105 | 0.090 | 0.120 |  | 1 |  | EQ-5D-3L | Instrument Defined |  | NO | Zimbabwe |
| 49 | Estimation of an Instrument-Defined Minimally Important Difference in EQ-5D-5L Index Scores Based on Scoring Algorithms Derived Using the EQ-VT Version 2 Valuation Protocols | Edward Henry | 0.149 | 0.121 | 0.177 |  | 1 |  | EQ-5D-3L | Instrument Defined |  | NO | Great Britain |
| 50 | Estimation of an Instrument-Defined Minimally Important Difference in EQ-5D-5L Index Scores Based on Scoring Algorithms Derived Using the EQ-VT Version 2 Valuation Protocols | Edward Henry | 0.106 | 0.087 | 0.125 |  | 1 |  | EQ-5D-3L | Instrument Defined |  | NO | United States |
| 51 | Responsiveness and minimal clinically important difference for the EQ-5D in chronic rhinosinusitis | Lloyd Hoehle | 0.04 | 0.01 | 0.08 | 0.34 | 1 | 203 | EQ-5D-3L | Mean | Chronic Rhinositis | NO | United States |
| 52 | Responsiveness and minimal clinically important difference for the EQ-5D in chronic rhinosinusitis | Lloyd Hoehle | 0.01 |  |  | 0.34 | 1 | 203 | EQ-5D-3L | ROC Curve | Chronic Rhinositis | NO | United States |
| 53 | Nephrectomy Complication is a Risk Factor of Clinically Meaningful Decrease in Health Utility Among Living Kidney Donors | Kossar Hosseini | 0.113 | 0.070 | 0.156 | 0.19 | 1 | 228 | EQ-5D-3L | Mean |  | YES | France |
| 54 | Nephrectomy Complication is a Risk Factor of Clinically Meaningful Decrease in Health Utility Among Living Kidney Donors | Kossar Hosseini | 0.116 | 0.077 | 0.154 | 0.25 | 1 | 216 | SF-6D.v1 | Mean |  | YES | France |
| 55 | The early outcome of surgical treatment for femoroacetabular impingement: success depends on how you measure it | Franco Impellizzeri | 0.16 |  |  |  | 1 | 102 | EQ-5D-3L | ROC Curve | Femoroacetabular Impingement | YES | Switzerland |
| 56 | Minimally important differences and predictors of change in quality of life in type 2 diabetes: A community-based survey in China | Xuejing Jin | 0.008 |  |  |  | 0 | 1958 | EQ-5D-3L | Mean | Type 2 Diabetes | NO | China |
| 57 | Minimally important differences and predictors of change in quality of life in type 2 diabetes: A community-based survey in China | Xuejing Jin | 0.049 |  |  |  | 0 | 1958 | EQ-5D-3L | Mean | Type 2 Diabetes | NO | China |
| 58 | Minimally important differences and predictors of change in quality of life in type 2 diabetes: A community-based survey in China | Xuejing Jin | 0.003 |  |  |  | 0 | 1958 | EQ-5D-3L | Mean | Type 2 Diabetes | NO | China |
| 59 | Minimally important differences and predictors of change in quality of life in type 2 diabetes: A community-based survey in China | Xuejing Jin | 0.077 |  |  |  | 0 | 1958 | EQ-5D-3L | Mean | Type 2 Diabetes | NO | China |
| 60 | Comparison of the SF6D, the EQ5D, and the oswestry disability index in patients with chronic low back pain and degenerative disk disease | Lars Johnsen | 0.031 |  |  | 0.76 | 1 | 113 | SF-6D.v1 | ROC Curve | Degenerative Disc Disease and Chronic Lower Back Pain | YES | Norway |
| 61 | Comparison of the SF6D, the EQ5D, and the oswestry disability index in patients with chronic low back pain and degenerative disk disease | Lars Johnsen | 0.173 |  |  | 0.55 | 1 | 113 | EQ-5D-3L | ROC Curve | Degenerative Disc Disease and Chronic Lower Back Pain | YES | Norway |
| 62 | Minimum clinically important difference in outcome scores among patients undergoing cervical laminoplasty | So Kato | 0.0485 |  |  |  | 1 | 101 | EQ-5D-3L | ROC Curve | Degenerative Cervical Myelopathy | YES | Japan |
| 63 | Reliability, validity, and minimally important differences of the SF-6D in systemic scelrosis | Dinesh Khanna | 0.035 |  |  |  | 1 | 127 | SF-6D.v1 | Mean | Systemic Sclerosis | NO | United States |
| 64 | Estimation of minimally important differences in the EQ-5D and SF-6D indices and their utility in stroke | Sang-Kyu Kim | 0.08 | -0.34 | 0.50 | 0.5 | 1 | 484 | EQ-5D-3L | Mean | Stroke | NO | South Korea |
| 65 | Estimation of minimally important differences in the EQ-5D and SF-6D indices and their utility in stroke | Sang-Kyu Kim | 0.12 | -0.4 | 0.64 | 0.5 | 1 | 484 | EQ-5D-3L | Mean | Stroke | NO | South Korea |
| 66 | Estimation of minimally important differences in the EQ-5D and SF-6D indices and their utility in stroke | Sang-Kyu Kim | 0.09 | -0.47 | 0.65 | 0.5 | 1 | 442 | EQ-5D-3L | Mean | Stroke | NO | South Korea |
| 67 | Estimation of minimally important differences in the EQ-5D and SF-6D indices and their utility in stroke | Sang-Kyu Kim | 0.12 | -0.34 | 0.58 | 0.5 | 1 | 442 | EQ-5D-3L | Mean | Stroke | NO | South Korea |
| 68 | Estimation of minimally important differences in the EQ-5D and SF-6D indices and their utility in stroke | Sang-Kyu Kim | 0.07 | -0.19 | 0.33 | 0.5 | 1 | 484 | SF-6D.v1 | Mean | Stroke | NO | South Korea |
| 69 | Estimation of minimally important differences in the EQ-5D and SF-6D indices and their utility in stroke | Sang-Kyu Kim | 0.04 | -0.2 | 0.28 | 0.5 | 1 | 484 | SF-6D.v1 | Mean | Stroke | NO | South Korea |
| 70 | Estimation of minimally important differences in the EQ-5D and SF-6D indices and their utility in stroke | Sang-Kyu Kim | 0.14 | -0.16 | 0.44 | 0.5 | 1 | 442 | SF-6D.v1 | Mean | Stroke | NO | South Korea |
| 71 | Estimation of minimally important differences in the EQ-5D and SF-6D indices and their utility in stroke | Sang-Kyu Kim | 0.04 | -0.22 | 0.30 | 0.5 | 1 | 442 | SF-6D.v1 | Mean | Stroke | NO | South Korea |
| 72 | Responsiveness and minimal important score differences in quality-of-life questionnaires: a comparison of the EORTC QLQ-C30 cancer specific questionnaire to the generic utility questionnaires EQ-5D and 15D in patients with multiple myeloma | Ann Kvam | 0.08 | 0.04 | 0.12 |  | 0 | 239 | EQ-5D-3L | Mean | Multiple Myeloma | NO | Norway |
| 73 | Responsiveness and minimal important score differences in quality-of-life questionnaires: a comparison of the EORTC QLQ-C30 cancer specific questionnaire to the generic utility questionnaires EQ-5D and 15D in patients with multiple myeloma | Ann Kvam | 0.10 | 0.04 | 0.16 |  | 0 | 239 | EQ-5D-3L | Mean | Multiple Myeloma | NO | Norway |
| 74 | Responsiveness and minimal important score differences in quality-of-life questionnaires: a comparison of the EORTC QLQ-C30 cancer specific questionnaire to the generic utility questionnaires EQ-5D and 15D in patients with multiple myeloma | Ann Kvam | 0.03 | 0.01 | 0.05 |  | 0 | 239 | 15-D | Mean | Multiple Myeloma | NO | Norway |
| 75 | Responsiveness and minimal important score differences in quality-of-life questionnaires: a comparison of the EORTC QLQ-C30 cancer specific questionnaire to the generic utility questionnaires EQ-5D and 15D in patients with multiple myeloma | Ann Kvam | 0.02 | -0.01 | 0.05 |  | 0 | 239 | 15-D | Mean | Multiple Myeloma | NO | Norway |
| 76 | Identification of Cutpoints for Acceptable Health Status and Important Improvement in Patient-Reported Outcomes, in Rheumatoid Arthritis, Psoriatic Arthritis and Ankylosing Spondylitis | Maria Kvamme | 0.10 |  |  | 0.34 | 0 | 728 | EQ-5D-3L | ROC Curve | Rheumatoid Arthritis | NO | Norway |
| 77 | Identification of Cutpoints for Acceptable Health Status and Important Improvement in Patient-Reported Outcomes, in Rheumatoid Arthritis, Psoriatic Arthritis and Ankylosing Spondylitis | Maria Kvamme | 0.18 |  |  | 0.34 | 0 | 250 | EQ-5D-3L | ROC Curve | Psoriatic Arthritis | NO | Norway |
| 78 | Identification of Cutpoints for Acceptable Health Status and Important Improvement in Patient-Reported Outcomes, in Rheumatoid Arthritis, Psoriatic Arthritis and Ankylosing Spondylitis | Maria Kvamme | 0.19 |  |  | 0.34 | 0 | 207 | EQ-5D-3L | ROC Curve | Ankylosing Spondylitis | NO | Norway |
| 79 | Identification of Cutpoints for Acceptable Health Status and Important Improvement in Patient-Reported Outcomes, in Rheumatoid Arthritis, Psoriatic Arthritis and Ankylosing Spondylitis | Maria Kvamme | 0.08 |  |  | 0.41 | 0 | 2771 | SF-6D.v1 | ROC Curve | Rheumatoid Arthritis | NO | Norway |
| 80 | Identification of Cutpoints for Acceptable Health Status and Important Improvement in Patient-Reported Outcomes, in Rheumatoid Arthritis, Psoriatic Arthritis and Ankylosing Spondylitis | Maria Kvamme | 0.07 |  |  | 0.41 | 0 | 819 | SF-6D.v1 | ROC Curve | Psoriatic Arthritis | NO | Norway |
| 81 | Identification of Cutpoints for Acceptable Health Status and Important Improvement in Patient-Reported Outcomes, in Rheumatoid Arthritis, Psoriatic Arthritis and Ankylosing Spondylitis | Maria Kvamme | 0.09 |  |  | 0.41 | 0 | 465 | SF-6D.v1 | ROC Curve | Ankylosing Spondylitis | NO | Norway |
| 82 | Identification of Cutpoints for Acceptable Health Status and Important Improvement in Patient-Reported Outcomes, in Rheumatoid Arthritis, Psoriatic Arthritis and Ankylosing Spondylitis | Maria Kvamme | 0.00 |  |  | 0.34 | 0 | 728 | EQ-5D-3L | Percentile | Rheumatoid Arthritis | NO | Norway |
| 83 | Identification of Cutpoints for Acceptable Health Status and Important Improvement in Patient-Reported Outcomes, in Rheumatoid Arthritis, Psoriatic Arthritis and Ankylosing Spondylitis | Maria Kvamme | 0.00 |  |  | 0.34 | 0 | 250 | EQ-5D-3L | Percentile | Psoriatic Arthritis | NO | Norway |
| 84 | Identification of Cutpoints for Acceptable Health Status and Important Improvement in Patient-Reported Outcomes, in Rheumatoid Arthritis, Psoriatic Arthritis and Ankylosing Spondylitis | Maria Kvamme | 0.04 |  |  | 0.34 | 0 | 207 | EQ-5D-3L | Percentile | Ankylosing Spondylitis | NO | Norway |
| 85 | Identification of Cutpoints for Acceptable Health Status and Important Improvement in Patient-Reported Outcomes, in Rheumatoid Arthritis, Psoriatic Arthritis and Ankylosing Spondylitis | Maria Kvamme | 0.02 |  |  | 0.41 | 0 | 2771 | SF-6D.v1 | Percentile | Rheumatoid Arthritis | NO | Norway |
| 86 | Identification of Cutpoints for Acceptable Health Status and Important Improvement in Patient-Reported Outcomes, in Rheumatoid Arthritis, Psoriatic Arthritis and Ankylosing Spondylitis | Maria Kvamme | 0.01 |  |  | 0.41 | 0 | 819 | SF-6D.v1 | Percentile | Psoriatic Arthritis | NO | Norway |
| 87 | Identification of Cutpoints for Acceptable Health Status and Important Improvement in Patient-Reported Outcomes, in Rheumatoid Arthritis, Psoriatic Arthritis and Ankylosing Spondylitis | Maria Kvamme | 0.05 |  |  | 0.41 | 0 | 465 | SF-6D.v1 | Percentile | Ankylosing Spondylitis | NO | Norway |
| 88 | A comparison of the measurement properties and estimation of minimal important differences of the EQ-5D and SF-6D utility measures in patients with systemic sclerosis | Linda Kwakkenbos | 0.05 | -0.01 | 0.11 |  | 1 | 148 | EQ-5D-3L | Mean | Systemic Sclerosis | NO | Netherlands |
| 89 | A comparison of the measurement properties and estimation of minimal important differences of the EQ-5D and SF-6D utility measures in patients with systemic sclerosis | Linda Kwakkenbos | 0.12 | 0.05 | 0.18 |  | 1 | 148 | EQ-5D-3L | Mean | Systemic Sclerosis | NO | Netherlands |
| 90 | A comparison of the measurement properties and estimation of minimal important differences of the EQ-5D and SF-6D utility measures in patients with systemic sclerosis | Linda Kwakkenbos | 0.10 | 0.01 | 0.18 | 0.63 | 1 | 148 | EQ-5D-3L | Mean | Systemic Sclerosis | NO | Netherlands |
| 91 | A comparison of the measurement properties and estimation of minimal important differences of the EQ-5D and SF-6D utility measures in patients with systemic sclerosis | Linda Kwakkenbos | 0.14 | 0.07 | 0.20 | 0.63 | 1 | 148 | EQ-5D-3L | Mean | Systemic Sclerosis | NO | Netherlands |
| 92 | A comparison of the measurement properties and estimation of minimal important differences of the EQ-5D and SF-6D utility measures in patients with systemic sclerosis | Linda Kwakkenbos | 0.05 | 0.00 | 0.09 |  | 1 | 147 | SF-6D.v1 | Mean | Systemic Sclerosis | NO | Netherlands |
| 93 | A comparison of the measurement properties and estimation of minimal important differences of the EQ-5D and SF-6D utility measures in patients with systemic sclerosis | Linda Kwakkenbos | 0.03 | 0.00 | 0.06 |  | 1 | 147 | SF-6D.v1 | Mean | Systemic Sclerosis | NO | Netherlands |
| 94 | A comparison of the measurement properties and estimation of minimal important differences of the EQ-5D and SF-6D utility measures in patients with systemic sclerosis | Linda Kwakkenbos | 0.05 | 0.01 | 0.09 | 0.63 | 1 | 147 | SF-6D.v1 | Mean | Systemic Sclerosis | NO | Netherlands |
| 95 | A comparison of the measurement properties and estimation of minimal important differences of the EQ-5D and SF-6D utility measures in patients with systemic sclerosis | Linda Kwakkenbos | 0.04 | 0.02 | 0.07 | 0.63 | 1 | 147 | SF-6D.v1 | Mean | Systemic Sclerosis | NO | Netherlands |
| 96 | Validity and responsiveness of the EQ-5D-5L and SF-6D in patients with health complaints attributed to their amalgam fillings: a prospective cohort study of patients undergoing amalgam removal | Admassu Lamu | 0.118 | 0.038 | 0.198 | 0.53 | 0 | 32 | EQ-5D-5L | Mean |  | NO | Norway |
| 97 | Validity and responsiveness of the EQ-5D-5L and SF-6D in patients with health complaints attributed to their amalgam fillings: a prospective cohort study of patients undergoing amalgam removal | Admassu Lamu | 0.064 | 0.029 | 0.099 | 0.45 | 0 | 32 | SF-6D.v1 | Mean |  | NO | Norway |
| 98 | Validity and responsiveness of the EQ-5D-5L and SF-6D in patients with health complaints attributed to their amalgam fillings: a prospective cohort study of patients undergoing amalgam removal | Admassu Lamu | 0.103 | 0.062 | 0.237 | 0.49 | 1 | 32 | EQ-5D-5L | Regression |  | NO | Norway |
| 99 | Validity and responsiveness of the EQ-5D-5L and SF-6D in patients with health complaints attributed to their amalgam fillings: a prospective cohort study of patients undergoing amalgam removal | Admassu Lamu | 0.056 | 0.02 | 0.143 | 0.51 | 1 | 32 | SF-6D.v1 | Regression |  | NO | Norway |
| 100 | Minimal clinically important differences for the EQ-5D and the QWB-SA in Post-traumatic Stress Disorder (PTSD): results from a Doubly Randomised Preference Trial (DRPT) | Quang Le | 0.05 | 0.03 | 0.07 | 0.37 | 1 | 155 | EQ-5D-3L | Regression | Post-Traumatic Stress Disorder | NO | United States |
| 101 | Minimal clinically important differences for the EQ-5D and the QWB-SA in Post-traumatic Stress Disorder (PTSD): results from a Doubly Randomised Preference Trial (DRPT) | Quang Le | 0.08 | 0.04 | 0.11 | 0.35 | 1 | 155 | EQ-5D-3L | Regression | Post-Traumatic Stress Disorder | NO | United States |
|  |  |  |  |  |  |  |  |  |  |  |  |  |  |
| 102 | Minimal clinically important differences for the EQ-5D and the QWB-SA in Post-traumatic Stress Disorder (PTSD): results from a Doubly Randomised Preference Trial (DRPT) | Quang Le | 0.05 |  |  | 0.44 | 1 | 155 | EQ-5D-3L | ROC Curve | Post-Traumatic Stress Disorder | NO | United States |
| 103 | Minimal clinically important differences for the EQ-5D and the QWB-SA in Post-traumatic Stress Disorder (PTSD): results from a Doubly Randomised Preference Trial (DRPT) | Quang Le | 0.03 | 0.02 | 0.05 | 0.39 | 1 | 155 | QWB-SA | Regression | Post-Traumatic Stress Disorder | NO | United States |
| 104 | Minimal clinically important differences for the EQ-5D and the QWB-SA in Post-traumatic Stress Disorder (PTSD): results from a Doubly Randomised Preference Trial (DRPT) | Quang Le | 0.05 | 0.03 | 0.08 | 0.41 | 1 | 155 | QWB-SA | Regression | Post-Traumatic Stress Disorder | NO | United States |
| 105 | Minimal clinically important differences for the EQ-5D and the QWB-SA in Post-traumatic Stress Disorder (PTSD): results from a Doubly Randomised Preference Trial (DRPT) | Quang Le | 0.03 |  |  | 0.43 | 1 | 155 | QWB-SA | ROC Curve | Post-Traumatic Stress Disorder | NO | United States |
| 106 | Validity, responsiveness, and minimal important difference for the SF-6D health utility scale in a spinal cord injured population | Bonsan Lee | 0.10 | -0.18 | 0.38 |  | 1 | 305 | SF-6D.v1 | Mean | Spinal Cord Injury with Neuropathic Bladder | NO | Australia |
| 107 | Validity, responsiveness, and minimal important difference for the SF-6D health utility scale in a spinal cord injured population | Bonsan Lee | 0.04 | -0.28 | 0.36 |  | 1 | 305 | SF-6D.v1 | Mean | Spinal Cord Injury with Neuropathic Bladder | NO | Australia |
| 108 | Using Instrument-Defined Health State Transitions to Estimate Minimally Important Differences for Four Preference-Based Health-Related Quality of Life Instruments | Nan Luo | 0.040 | -0.012 | 0.092 |  | 1 |  | EQ-5D-3L | Instrument Defined |  | NO | Singapore |
| 109 | Using Instrument-Defined Health State Transitions to Estimate Minimally Important Differences for Four Preference-Based Health-Related Quality of Life Instruments | Nan Luo | 0.082 | 0.018 | 0.146 |  | 1 |  | EQ-5D-3L | Instrument Defined |  | NO | Singapore |
| 110 | Using Instrument-Defined Health State Transitions to Estimate Minimally Important Differences for Four Preference-Based Health-Related Quality of Life Instruments | Nan Luo | 0.045 | -0.033 | 0.123 |  | 1 |  | HUI2 | Instrument Defined |  | NO | Singapore |
| 111 | Using Instrument-Defined Health State Transitions to Estimate Minimally Important Differences for Four Preference-Based Health-Related Quality of Life Instruments | Nan Luo | 0.032 | -0.022 | 0.086 |  | 1 |  | HUI3 | Instrument Defined |  | NO | Singapore |
| 112 | Using Instrument-Defined Health State Transitions to Estimate Minimally Important Differences for Four Preference-Based Health-Related Quality of Life Instruments | Nan Luo | 0.027 | -0.029 | 0.083 |  | 1 |  | SF-6D.v1 | Instrument Defined |  | NO | Singapore |
| 113 | Validity, responsiveness and minimal important change of the EQ-5D-5L in patients after rotator cuff repair, shoulder arthroplasty or thumb carpometacarpal arthroplasty | Miriam Marks | 0.048 |  |  |  | 1 | 148 | EQ-5D-5L | Difference of Means | Rotator Cuff Tear | YES | Switzerland |
| 114 | Validity, responsiveness and minimal important change of the EQ-5D-5L in patients after rotator cuff repair, shoulder arthroplasty or thumb carpometacarpal arthroplasty | Miriam Marks | 0.094 |  |  |  | 1 | 148 | EQ-5D-5L | Difference of Means | Glenohumeral Osteoarthritis | YES | Switzerland |
| 115 | Validity, responsiveness and minimal important change of the EQ-5D-5L in patients after rotator cuff repair, shoulder arthroplasty or thumb carpometacarpal arthroplasty | Miriam Marks | 0.027 |  |  |  | 1 | 150 | EQ-5D-5L | Difference of Means | Thumb Carpometacarpal Osteoarthritis | YES | Switzerland |
| 116 | Validity, responsiveness and minimal important change of the EQ-5D-5L in patients after rotator cuff repair, shoulder arthroplasty or thumb carpometacarpal arthroplasty | Miriam Marks | 0.161 |  |  |  | 1 | 148 | EQ-5D-5L | Mean | Rotator Cuff Tear | YES | Switzerland |
| 117 | Validity, responsiveness and minimal important change of the EQ-5D-5L in patients after rotator cuff repair, shoulder arthroplasty or thumb carpometacarpal arthroplasty | Miriam Marks | 0.183 |  |  |  | 1 | 148 | EQ-5D-5L | Mean | Glenohumeral Osteoarthritis | YES | Switzerland |
| 118 | Validity, responsiveness and minimal important change of the EQ-5D-5L in patients after rotator cuff repair, shoulder arthroplasty or thumb carpometacarpal arthroplasty | Miriam Marks | 0.209 |  |  |  | 1 | 150 | EQ-5D-5L | Mean | Thumb Carpometacarpal Osteoarthritis | YES | Switzerland |
| 119 | Validity, responsiveness and minimal important change of the EQ-5D-5L in patients after rotator cuff repair, shoulder arthroplasty or thumb carpometacarpal arthroplasty | Miriam Marks | 0.130 |  |  |  | 1 | 148 | EQ-5D-5L | ROC Curve | Rotator Cuff Tear | YES | Switzerland |
| 120 | Validity, responsiveness and minimal important change of the EQ-5D-5L in patients after rotator cuff repair, shoulder arthroplasty or thumb carpometacarpal arthroplasty | Miriam Marks | 0.058 |  |  |  | 1 | 148 | EQ-5D-5L | ROC Curve | Glenohumeral Osteoarthritis | YES | Switzerland |
| 121 | Validity, responsiveness and minimal important change of the EQ-5D-5L in patients after rotator cuff repair, shoulder arthroplasty or thumb carpometacarpal arthroplasty | Miriam Marks | 0.166 |  |  |  | 1 | 150 | EQ-5D-5L | ROC Curve | Thumb Carpometacarpal Osteoarthritis | YES | Switzerland |
| 122 | A comparison of generic, indirect utility measures (the HUI2, HUI3, SF-6D, and the EQ-5D) and disease-specific instruments (the RAQoL and the HAQ) in rheumatoid arthritis | Carlo Marra | 0.04 |  |  | 0.66 | 1 | 304 | HUI2 | Regression | Rheumatoid Arthritis | NO | Canada |
| 123 | A comparison of generic, indirect utility measures (the HUI2, HUI3, SF-6D, and the EQ-5D) and disease-specific instruments (the RAQoL and the HAQ) in rheumatoid arthritis | Carlo Marra | 0.07 |  |  | 0.76 | 1 | 303 | HUI3 | Regression | Rheumatoid Arthritis | NO | Canada |
| 124 | A comparison of generic, indirect utility measures (the HUI2, HUI3, SF-6D, and the EQ-5D) and disease-specific instruments (the RAQoL and the HAQ) in rheumatoid arthritis | Carlo Marra | 0.03 |  |  | 0.73 | 1 | 302 | SF-6D.v1 | Regression | Rheumatoid Arthritis | NO | Canada |
| 125 | A comparison of generic, indirect utility measures (the HUI2, HUI3, SF-6D, and the EQ-5D) and disease-specific instruments (the RAQoL and the HAQ) in rheumatoid arthritis | Carlo Marra | 0.05 |  |  | 0.61 | 1 | 308 | EQ-5D-3L | Regression | Rheumatoid Arthritis | NO | Canada |
| 126 | Measurement properties of the EuroQoL EQ-5D-5L to assess quality of life in patients undergoing carpal tunnel release | C Marti | 0.09 | 0.02 | 0.16 |  | 1 | 56 | EQ-5D-5L | Mean | Carpal Tunnel Syndrome | YES | Switzerland |
| 127 | Instrument Defined Estimates of the Minimally Important Difference for the EQ-5D-5L Index Scores | Nathan McClure | 0.056 | 0.034 | 0.078 |  | 1 |  | EQ-5D-5L | Instrument Defined |  | NO | Canada |
| 128 | Instrument Defined Estimates of the Minimally Important Difference for the EQ-5D-5L Index Scores | Nathan McClure | 0.069 | 0.055 | 0.083 |  | 1 |  | EQ-5D-5L | Instrument Defined |  | NO | Canada |
| 129 | Instrument Defined Estimates of the Minimally Important Difference for the EQ-5D-5L Index Scores | Nathan McClure | 0.061 | 0.045 | 0.077 |  | 1 |  | EQ-5D-5L | Instrument Defined |  | NO | Canada |
| 130 | Instrument Defined Estimates of the Minimally Important Difference for the EQ-5D-5L Index Scores | Nathan McClure | 0.048 | 0.040 | 0.056 |  | 1 |  | EQ-5D-5L | Instrument Defined |  | NO | Canada |
| 131 | Instrument Defined Estimates of the Minimally Important Difference for the EQ-5D-5L Index Scores | Nathan McClure | 0.063 | 0.037 | 0.089 |  | 1 |  | EQ-5D-5L | Instrument Defined |  | NO | Canada |
| 132 | Instrument Defined Estimates of the Minimally Important Difference for the EQ-5D-5L Index Scores | Nathan McClure | 0.063 | 0.025 | 0.101 |  | 1 |  | EQ-5D-5L | Instrument Defined |  | NO | Canada |
| 133 | Instrument Defined Estimates of the Minimally Important Difference for the EQ-5D-5L Index Scores | Nathan McClure | 0.037 | 0.035 | 0.039 |  | 1 |  | EQ-5D-5L | Instrument Defined |  | NO | Canada |
| 134 | Instrument Defined Estimates of the Minimally Important Difference for the EQ-5D-5L Index Scores | Nathan McClure | 0.058 | 0.048 | 0.068 |  | 1 |  | EQ-5D-5L | Instrument Defined |  | NO | Canada |
| 135 | Instrument Defined Estimates of the Minimally Important Difference for the EQ-5D-5L Index Scores | Nathan McClure | 0.045 | 0.027 | 0.063 |  | 1 |  | EQ-5D-5L | Instrument Defined |  | NO | Canada |
| 136 | Instrument Defined Estimates of the Minimally Important Difference for the EQ-5D-5L Index Scores | Nathan McClure | 0.044 | 0.036 | 0.052 |  | 1 |  | EQ-5D-5L | Instrument Defined |  | NO | Canada |
| 137 | Instrument Defined Estimates of the Minimally Important Difference for the EQ-5D-5L Index Scores | Nathan McClure | 0.037 | 0.021 | 0.053 |  | 1 |  | EQ-5D-5L | Instrument Defined |  | NO | Canada |
| 138 | Instrument Defined Estimates of the Minimally Important Difference for the EQ-5D-5L Index Scores | Nathan McClure | 0.040 | 0.020 | 0.060 |  | 1 |  | EQ-5D-5L | Instrument Defined |  | NO | Canada |
| 139 | Minimally Important Difference of the EQ-5D-5L Index Score in Adults with Type 2 Diabetes | Nathan McClure | 0.049 | 0.048 | 0.049 |  | 1 | 1927 | EQ-5D-5L | Instrument Defined | Type 2 Diabetes | NO | Canada |
| 140 | Minimally Important Difference of the EQ-5D-5L Index Score in Adults with Type 2 Diabetes | Nathan McClure | 0.037 | 0.037 | 0.037 |  | 1 | 1927 | EQ-5D-5L | Instrument Defined | Type 2 Diabetes | NO | Canada |
| 141 | Minimally Important Difference of the EQ-5D-5L Index Score in Adults with Type 2 Diabetes | Nathan McClure | 0.043 | 0.043 | 0.044 |  | 1 | 1927 | EQ-5D-5L | Instrument Defined | Type 2 Diabetes | NO | Canada |
| 142 | Minimally Important Difference of the EQ-5D-5L Index Score in Adults with Type 2 Diabetes | Nathan McClure | 0.038 | 0.037 | 0.038 |  | 1 | 1927 | EQ-5D-5L | Instrument Defined | Type 2 Diabetes | NO | Canada |
| 143 | Minimally Important Difference of the EQ-5D-5L Index Score in Adults with Type 2 Diabetes | Nathan McClure | 0.053 | 0.052 | 0.054 |  | 1 | 1927 | EQ-5D-5L | Instrument Defined | Type 2 Diabetes | NO | Canada |
| 144 | Minimally Important Difference of the EQ-5D-5L Index Score in Adults with Type 2 Diabetes | Nathan McClure | 0.038 | 0.037 | 0.038 |  | 1 | 1927 | EQ-5D-5L | Instrument Defined | Type 2 Diabetes | NO | Canada |
| 145 | Minimally Important Difference of the EQ-5D-5L Index Score in Adults with Type 2 Diabetes | Nathan McClure | 0.042 | 0.030 | 0.055 |  | 0 | 295 | EQ-5D-5L | Regression | Type 2 Diabetes | NO | Canada |
| 146 | Minimally Important Difference of the EQ-5D-5L Index Score in Adults with Type 2 Diabetes | Nathan McClure | 0.035 | 0.017 | 0.053 |  | 0 | 138 | EQ-5D-5L | Regression | Type 2 Diabetes | NO | Canada |
| 147 | Minimally Important Difference of the EQ-5D-5L Index Score in Adults with Type 2 Diabetes | Nathan McClure | 0.049 | 0.032 | 0.067 |  | 0 | 157 | EQ-5D-5L | Regression | Type 2 Diabetes | NO | Canada |
| 148 | A Longitudinal Comparison of Five Preference-weighted Health State Classification Systems in Persons with Intervertebral Disc Herniation | Christine McDonough | 0.08 | 0.06 | 0.11 |  | 1 | 172 | EQ-5D-3L | Mean | Intervertebral Disc Herniation | YES | United States |
| 149 | A Longitudinal Comparison of Five Preference-weighted Health State Classification Systems in Persons with Intervertebral Disc Herniation | Christine McDonough | 0.17 | 0.13 | 0.20 |  | 1 | 170 | EQ-5D-3L | Mean | Intervertebral Disc Herniation | YES | United States |
| 150 | A Longitudinal Comparison of Five Preference-weighted Health State Classification Systems in Persons with Intervertebral Disc Herniation | Christine McDonough | 0.10 | 0.07 | 0.13 |  | 1 | 194 | EQ-5D-3L | Mean | Intervertebral Disc Herniation | YES | United States |
| 151 | A Longitudinal Comparison of Five Preference-weighted Health State Classification Systems in Persons with Intervertebral Disc Herniation | Christine McDonough | 0.10 | 0.06 | 0.13 |  | 1 | 403 | EQ-5D-3L | Mean | Intervertebral Disc Herniation | YES | United States |
| 152 | A Longitudinal Comparison of Five Preference-weighted Health State Classification Systems in Persons with Intervertebral Disc Herniation | Christine McDonough | 0.11 | 0.08 | 0.15 |  | 1 | 172 | HUI3 | Mean | Intervertebral Disc Herniation | YES | United States |
| 153 | A Longitudinal Comparison of Five Preference-weighted Health State Classification Systems in Persons with Intervertebral Disc Herniation | Christine McDonough | 0.21 | 0.16 | 0.25 |  | 1 | 170 | HUI3 | Mean | Intervertebral Disc Herniation | YES | United States |
| 154 | A Longitudinal Comparison of Five Preference-weighted Health State Classification Systems in Persons with Intervertebral Disc Herniation | Christine McDonough | 0.15 | 0.11 | 0.19 |  | 1 | 194 | HUI3 | Mean | Intervertebral Disc Herniation | YES | United States |
| 155 | A Longitudinal Comparison of Five Preference-weighted Health State Classification Systems in Persons with Intervertebral Disc Herniation | Christine McDonough | 0.13 | 0.09 | 0.18 |  | 1 | 403 | HUI3 | Mean | Intervertebral Disc Herniation | YES | United States |
| 156 | A Longitudinal Comparison of Five Preference-weighted Health State Classification Systems in Persons with Intervertebral Disc Herniation | Christine McDonough | 0.09 | 0.07 | 0.12 |  | 1 | 172 | HUI2 | Mean | Intervertebral Disc Herniation | YES | United States |
| 157 | A Longitudinal Comparison of Five Preference-weighted Health State Classification Systems in Persons with Intervertebral Disc Herniation | Christine McDonough | 0.18 | 0.15 | 0.21 |  | 1 | 170 | HUI2 | Mean | Intervertebral Disc Herniation | YES | United States |
| 158 | A Longitudinal Comparison of Five Preference-weighted Health State Classification Systems in Persons with Intervertebral Disc Herniation | Christine McDonough | 0.11 | 0.08 | 0.14 |  | 1 | 194 | HUI2 | Mean | Intervertebral Disc Herniation | YES | United States |
| 159 | A Longitudinal Comparison of Five Preference-weighted Health State Classification Systems in Persons with Intervertebral Disc Herniation | Christine McDonough | 0.09 | 0.05 | 0.12 |  | 1 | 403 | HUI2 | Mean | Intervertebral Disc Herniation | YES | United States |
| 160 | A Longitudinal Comparison of Five Preference-weighted Health State Classification Systems in Persons with Intervertebral Disc Herniation | Christine McDonough | 0.09 | 0.07 | 0.11 |  | 1 | 172 | SF-6D.v1 | Mean | Intervertebral Disc Herniation | YES | United States |
| 161 | A Longitudinal Comparison of Five Preference-weighted Health State Classification Systems in Persons with Intervertebral Disc Herniation | Christine McDonough | 0.11 | 0.09 | 0.13 |  | 1 | 170 | SF-6D.v1 | Mean | Intervertebral Disc Herniation | YES | United States |
| 162 | A Longitudinal Comparison of Five Preference-weighted Health State Classification Systems in Persons with Intervertebral Disc Herniation | Christine McDonough | 0.08 | 0.06 | 0.10 |  | 1 | 194 | SF-6D.v1 | Mean | Intervertebral Disc Herniation | YES | United States |
| 163 | A Longitudinal Comparison of Five Preference-weighted Health State Classification Systems in Persons with Intervertebral Disc Herniation | Christine McDonough | 0.08 | 0.06 | 0.11 |  | 1 | 403 | SF-6D.v1 | Mean | Intervertebral Disc Herniation | YES | United States |
| 164 | A Longitudinal Comparison of Five Preference-weighted Health State Classification Systems in Persons with Intervertebral Disc Herniation | Christine McDonough | 0.05 | 0.04 | 0.06 |  | 1 | 172 | eQWB | Mean | Intervertebral Disc Herniation | YES | United States |
| 165 | A Longitudinal Comparison of Five Preference-weighted Health State Classification Systems in Persons with Intervertebral Disc Herniation | Christine McDonough | 0.06 | 0.05 | 0.07 |  | 1 | 170 | eQWB | Mean | Intervertebral Disc Herniation | YES | United States |
| 166 | A Longitudinal Comparison of Five Preference-weighted Health State Classification Systems in Persons with Intervertebral Disc Herniation | Christine McDonough | 0.03 | 0.02 | 0.04 |  | 1 | 194 | eQWB | Mean | Intervertebral Disc Herniation | YES | United States |
| 167 | A Longitudinal Comparison of Five Preference-weighted Health State Classification Systems in Persons with Intervertebral Disc Herniation | Christine McDonough | 0.05 | 0.04 | 0.07 |  | 1 | 403 | eQWB | Mean | Intervertebral Disc Herniation | YES | United States |
| 168 | Comparing 15D Valuation Studies in Norway and Finland - Challenges When Combining Information from Several Valuation Tasks | Yvonne Michel | 0.0060 |  |  |  | 1 |  | 15D | Instrument Defined |  | NO | Norway |
| 169 | Defining Clinically Meaningful Thresholds for Patient-Reported Outcomes in Knee Arthroplasty | Jasper Most | 0.037 | 0.035 | 0.039 |  | 1 | 22696 | EQ-5D-3L | Regression |  | YES | Netherlands |
| 170 | Defining Clinically Meaningful Thresholds for Patient-Reported Outcomes in Knee Arthroplasty | Jasper Most | 0.101 |  |  |  | 1 | 22696 | EQ-5D-3L | Regression |  | YES | Netherlands |
| 171 | Investigating the minimally important difference of the Diabetes Health Profile (DHP-18) and the EQ-5D and SF-6D in a UK diabetes mellitus population | Brendan Mulhern | 0.058 | 0.048 | 0.069 | 0.30 | 0 | 1092 | EQ-5D-3L | Difference of Means | Diabetes Mellitus | NO | Great Britain |
| 172 | Investigating the minimally important difference of the Diabetes Health Profile (DHP-18) and the EQ-5D and SF-6D in a UK diabetes mellitus population | Brendan Mulhern | 0.038 | 0.034 | 0.042 | 0.31 | 0 | 1092 | SF-6D.v1 | Difference of Means | Diabetes Mellitus | NO | Great Britain |
| 173 | Minimal clinically important difference in patients who underwent decompression alone for lumbar degenerative disease | Hiroyuki Nakarai | 0.18 |  |  |  | 1 | 422 | EQ-5D-3L | ROC Curve | Degenerative Lumbar Spinal Stenosis | YES | Japan |
| 174 | Minimal clinically important difference in patients who underwent decompression alone for lumbar degenerative disease | Hiroyuki Nakarai | 0.13 | 0.08 | 0.18 |  | 1 | 422 | EQ-5D-3L | Mean | Degenerative Lumbar Spinal Stenosis | YES | Japan |
| 175 | Minimal clinically important difference in patients who underwent decompression alone for lumbar degenerative disease | Hiroyuki Nakarai | 0.07 |  |  |  | 1 | 422 | EQ-5D-3L | ROC Curve | Degenerative Lumbar Spinal Stenosis | YES | Japan |
| 176 | Minimal clinically important difference in patients who underwent decompression alone for lumbar degenerative disease | Hiroyuki Nakarai | 0.14 | 0.04 | 0.24 |  | 1 | 422 | EQ-5D-3L | Mean | Degenerative Lumbar Spinal Stenosis | YES | Japan |
| 177 | Minimal clinically important difference in patients who underwent decompression alone for lumbar degenerative disease | Hiroyuki Nakarai | 0.18 |  |  |  | 1 | 422 | EQ-5D-3L | ROC Curve | Degenerative Lumbar Spinal Stenosis | YES | Japan |
| 178 | Minimal clinically important difference in patients who underwent decompression alone for lumbar degenerative disease | Hiroyuki Nakarai | 0.12 | 0.06 | 0.18 |  | 1 | 422 | EQ-5D-3L | Mean | Degenerative Lumbar Spinal Stenosis | YES | Japan |
| 179 | Separating gains and losses in health when calculating the minimum important difference for mapped utility measures | Michael Nichol | 0.03 |  |  |  | 1 | 6932 | SF-6D.v1 | Mean |  | NO | United States |
| 180 | Separating gains and losses in health when calculating the minimum important difference for mapped utility measures | Michael Nichol | 0.02 |  |  |  | 1 | 6932 | SF-6D.v1 | Mean |  | NO | United States |
| 181 | Separating gains and losses in health when calculating the minimum important difference for mapped utility measures | Michael Nichol | 0.06 |  |  |  | 1 | 6932 | SF-6D.v1 | Mean |  | NO | United States |
| 182 | Separating gains and losses in health when calculating the minimum important difference for mapped utility measures | Michael Nichol | 0.03 |  |  |  | 1 | 6932 | HUI2 | Mean |  | NO | United States |
| 183 | Separating gains and losses in health when calculating the minimum important difference for mapped utility measures | Michael Nichol | 0.02 |  |  |  | 1 | 6932 | HUI2 | Mean |  | NO | United States |
| 184 | Separating gains and losses in health when calculating the minimum important difference for mapped utility measures | Michael Nichol | 0.05 |  |  |  | 1 | 6932 | HUI2 | Mean |  | NO | United States |
| 185 | The EQ-5D-5: health status questionnaire in COPD: validity, responsiveness and minimum important difference | Claire Nolan | 0.054 |  |  |  | 1 | 324 | EQ-5D-5L | Regression | Chronic Obstructive Pulmonary Disorder | NO | Great Britain |
| 186 | The EQ-5D-5: health status questionnaire in COPD: validity, responsiveness and minimum important difference | Claire Nolan | 0.059 |  |  | 0.38 | 1 | 324 | EQ-5D-5L | Regression | Chronic Obstructive Pulmonary Disorder | NO | Great Britain |
| 187 | The EQ-5D-5: health status questionnaire in COPD: validity, responsiveness and minimum important difference | Claire Nolan | 0.037 |  |  | 0.38 | 1 | 324 | EQ-5D-5L | ROC Curve | Chronic Obstructive Pulmonary Disorder | NO | Great Britain |
| 188 | ISSLS prize in clinical science 2020: the reliability and interpretability of score change in lumbar spine research. | Catharina Parai | 0.104 |  |  | 0.805 | 0 | 32935 | EQ-5D-3L | ROC Curve | Lumbar Disk Herniation, Lumbar Spinal Stenosis, or Degenerative Disk Disease | YES | Sweden |
| 189 | ISSLS prize in clinical science 2020: the reliability and interpretability of score change in lumbar spine research. | Catharina Parai | 0.18 |  |  | 0.805 | 0 | 9214 | EQ-5D-3L | ROC Curve | Lumbar Disk Herniation | YES | Sweden |
| 190 | ISSLS prize in clinical science 2020: the reliability and interpretability of score change in lumbar spine research. | Catharina Parai | 0.10 |  |  | 0.805 | 0 | 19252 | EQ-5D-3L | ROC Curve | Lumbar Spinal Stenosis | YES | Sweden |
| 191 | ISSLS prize in clinical science 2020: the reliability and interpretability of score change in lumbar spine research. | Catharina Parai | 0.10 |  |  | 0.805 | 0 | 5930 | EQ-5D-3L | ROC Curve | Degenerative Disk Disease | YES | Sweden |
| 192 | Minimal clinically important improvement (MCII) and patient acceptable symptom state (PASS) in total hip arthroplasty (THA) patients 1 year postoperatively | Aksel Paulsen | 0.14 | 0.10 | 0.18 | 0.27 | 1 | 1239 | EQ-5D-3L | Mean |  | YES | Denmark |
| 193 | Minimal clinically important improvement (MCII) and patient acceptable symptom state (PASS) in total hip arthroplasty (THA) patients 1 year postoperatively | Aksel Paulsen | 0.27 | 0.13 | 0.40 | 0.27 | 1 | 1239 | EQ-5D-3L | Percentile |  | YES | Denmark |
| 194 | Minimal clinically important improvement (MCII) and patient acceptable symptom state (PASS) in total hip arthroplasty (THA) patients 1 year postoperatively | Aksel Paulsen | 0.33 | 0.13 | 0.33 | 0.27 | 1 | 1239 | EQ-5D-3L | ROC Curve |  | YES | Denmark |
| 195 | Minimal clinically important improvement (MCII) and patient acceptable symptom state (PASS) in total hip arthroplasty (THA) patients 1 year postoperatively | Aksel Paulsen | 0.31 | 0.29 | 0.34 | 0.27 | 1 | 1239 | EQ-5D-3L | Mean |  | YES | Denmark |
| 196 | Minimal clinically important improvement (MCII) and patient acceptable symptom state (PASS) in total hip arthroplasty (THA) patients 1 year postoperatively | Aksel Paulsen | 0.40 | 0.35 | 0.45 | 0.27 | 1 | 1239 | EQ-5D-3L | Percentile |  | YES | Denmark |
| 197 | Minimal clinically important improvement (MCII) and patient acceptable symptom state (PASS) in total hip arthroplasty (THA) patients 1 year postoperatively | Aksel Paulsen | 0.41 | 0.37 | 0.44 | 0.27 | 1 | 1239 | EQ-5D-3L | ROC Curve |  | YES | Denmark |
| 198 | Applied Machine Learning for Spine Surgeons: Predicting Outcome for Patients Undergoing Treatment for Lumbar Disc Herniation Using PRO Data | Casper Pedersen | 0.17 |  |  |  | 1 | 1988 | EQ-5D-3L | ROC Curve | Lumbar Disc Herniation | YES | Denmark |
| 199 | Estimation of minimally important differences in EQ-5D utility and VAS scores in cancer | A. Simon Pickard | 0.09 |  |  |  | 1 | 534 | EQ-5D-3L | Difference of Means | Advanced Cancer | YES | United States |
| 200 | Estimation of minimally important differences in EQ-5D utility and VAS scores in cancer | A. Simon Pickard | 0.06 |  |  |  | 1 | 534 | EQ-5D-3L | Difference of Means | Advanced Cancer | YES | United States |
| 201 | Quality of life assessed with EQ-5D in patients undergoing glioma surgery: What is the responsiveness and minimal clinically important difference? | Lisa Sagberg | 0.15 |  |  |  | 1 | 142 | EQ-5D-3L | Regression | Supratentorial Glioma | YES | Norway |
| 202 | Quality of life assessed with EQ-5D in patients undergoing glioma surgery: What is the responsiveness and minimal clinically important difference? | Lisa Sagberg | 0.14 |  |  |  | 1 | 164 | EQ-5D-3L | Difference of Means | Supratentorial Glioma | YES | Norway |
| 203 | The validity and responsiveness of three quality of life measures in the assessment of psoriasis patients: results of a phase II study | Richard Shikiar | 0.10 | -0.38 | 0.58 | 0.57 | 1 | 147 | EQ-5D-3L | Mean | Plaque Psoriasis | NO | United States |
| 204 | The validity and responsiveness of three quality of life measures in the assessment of psoriasis patients: results of a phase II study | Richard Shikiar | 0.20 | -0.22 | 0.62 | 0.57 | 1 | 147 | EQ-5D-3L | Mean | Plaque Psoriasis | NO | United States |
| 205 | The validity and responsiveness of three quality of life measures in the assessment of psoriasis patients: results of a phase II study | Richard Shikiar | 0.09 |  |  | 0.44 | 1 | 147 | EQ-5D-3L | Difference of Means | Plaque Psoriasis | NO | United States |
| 206 | Japanese population norms for preference-based measures: EQ-5D-3L, EQ-5D-5L, and SF-6D | Takeru Shiroirwa | 0.064 |  |  |  | 0 | 1143 | EQ-5D-3L | Difference of Means |  | NO | Japan |
| 207 | Japanese population norms for preference-based measures: EQ-5D-3L, EQ-5D-5L, and SF-6D | Takeru Shiroirwa | 0.061 |  |  |  | 0 | 1143 | EQ-5D-5L | Difference of Means |  | NO | Japan |
| 208 | Japanese population norms for preference-based measures: EQ-5D-3L, EQ-5D-5L, and SF-6D | Takeru Shiroirwa | 0.073 |  |  |  | 0 | 1143 | SF-6D.v1 | Difference of Means |  | NO | Japan |
| 209 | Japanese population norms for preference-based measures: EQ-5D-3L, EQ-5D-5L, and SF-6D | Takeru Shiroirwa | 0.093 |  |  |  | 0 | 1143 | EQ-5D-3L | Difference of Means |  | NO | Japan |
| 210 | Japanese population norms for preference-based measures: EQ-5D-3L, EQ-5D-5L, and SF-6D | Takeru Shiroirwa | 0.093 |  |  |  | 0 | 1143 | EQ-5D-5L | Difference of Means |  | NO | Japan |
| 211 | Japanese population norms for preference-based measures: EQ-5D-3L, EQ-5D-5L, and SF-6D | Takeru Shiroirwa | 0.112 |  |  |  | 0 | 1143 | SF-6D.v1 | Difference of Means |  | NO | Japan |
| 212 | Responsiveness of outcome measures in patients with superior labral anterior and posterior lesions | Øystein Skare | 0.53 |  |  |  | 0 | 89 | EQ-5D-3L | ROC Curve | Superior Labral Anterior and Posterior Lesions | YES | Norway |
| 213 | Validity, Reliability, and Responsiveness of the EQ-5D in Inflammatory Bowel Disease in Germany | Renee Stark | 0.050 | 0.012 | 0.088 |  | 0 | 424 | EQ-5D-3L | Regression | Crohn's Disease and Ulcerative Colitis | NO | Germany |
| 214 | Validity, Reliability, and Responsiveness of the EQ-5D in Inflammatory Bowel Disease in Germany | Renee Stark | 0.067 | 0.021 | 0.113 |  | 0 | 424 | EQ-5D-3L | Regression | Crohn's Disease and Ulcerative Colitis | NO | Germany |
| 215 | Validity, Reliability, and Responsiveness of the EQ-5D in Inflammatory Bowel Disease in Germany | Renee Stark | 0.066 | 0.007 | 0.125 |  | 0 | 379 | EQ-5D-3L | Regression | Crohn's Disease and Ulcerative Colitis (Active Disease) | NO | Germany |
| 216 | Validity, Reliability, and Responsiveness of the EQ-5D in Inflammatory Bowel Disease in Germany | Renee Stark | 0.042 | -0.034 | 0.118 |  | 0 | 379 | EQ-5D-3L | Regression | Crohn's Disease and Ulcerative Colitis (Remission) | NO | Germany |
| 217 | Validity, Reliability, and Responsiveness of the EQ-5D in Inflammatory Bowel Disease in Germany | Renee Stark | 0.093 | 0.042 | 0.144 |  | 0 | 379 | EQ-5D-3L | Regression | Crohn's Disease and Ulcerative Colitis (Active Disease) | NO | Germany |
| 218 | Validity, Reliability, and Responsiveness of the EQ-5D in Inflammatory Bowel Disease in Germany | Renee Stark | 0.038 | -0.014 | 0.090 |  | 0 | 379 | EQ-5D-3L | Regression | Crohn's Disease and Ulcerative Colitis (Remission) | NO | Germany |
| 219 | Assessment of the minimum clinically important difference in quality of life in schizophrenia measured by the Quality of Well-Being Scale and disease-specific measures | Soe Soe Thwin | 0.17 |  |  | 0.20 | 0 | 255 | QWB-SA | Equipercentile | Schizophrenia | NO | United States |
| 220 | Assessment of the minimum clinically important difference in quality of life in schizophrenia measured by the Quality of Well-Being Scale and disease-specific measures | Soe Soe Thwin | 0.21 |  |  | 0.20 | 0 | 255 | QWB-SA | Equipercentile | Schizophrenia | NO | United States |
| 221 | Assessment of the minimum clinically important difference in quality of life in schizophrenia measured by the Quality of Well-Being Scale and disease-specific measures | Soe Soe Thwin | 0.12 |  |  | 0.20 | 1 | 255 | QWB-SA | Equipercentile | Schizophrenia | NO | United States |
| 222 | Assessment of the minimum clinically important difference in quality of life in schizophrenia measured by the Quality of Well-Being Scale and disease-specific measures | Soe Soe Thwin | 0.25 |  |  | 0.20 | 1 | 255 | QWB-SA | Equipercentile | Schizophrenia | NO | United States |
| 223 | Minimum important difference of the EQ-­5D-­5L and EQ-­VAS in fibrotic interstitial lung disease | Amy Tsai | 0.044 |  |  |  | 1 | 1816 | EQ-5D-5L | Regression | Fibrotic Interstitial Lung Disease | NO | Canada |
| 224 | Minimum important difference of the EQ-­5D-­5L and EQ-­VAS in fibrotic interstitial lung disease | Amy Tsai | 0.024 |  |  |  | 1 | 1816 | EQ-5D-5L | Regression | Fibrotic Interstitial Lung Disease | NO | Canada |
| 225 | Minimum important difference of the EQ-­5D-­5L and EQ-­VAS in fibrotic interstitial lung disease | Amy Tsai | 0.010 |  |  |  | 1 | 1816 | EQ-5D-5L | Regression | Fibrotic Interstitial Lung Disease | NO | Canada |
| 226 | Minimum important difference of the EQ-­5D-­5L and EQ-­VAS in fibrotic interstitial lung disease | Amy Tsai | 0.027 |  |  |  | 1 | 1816 | EQ-5D-5L | Regression | Fibrotic Interstitial Lung Disease | NO | Canada |
| 227 | Minimum important difference of the EQ-­5D-­5L and EQ-­VAS in fibrotic interstitial lung disease | Amy Tsai | 0.013 |  |  |  | 1 | 1816 | EQ-5D-5L | Regression | Fibrotic Interstitial Lung Disease | NO | Canada |
| 228 | EQ-5D-derived health utilities and minimally important differences for chronic health conditions: 2011 Commonwealth Fund Survey of Sicker Adults in Canada | Kate Tsiplova | 0.044 | 0.025 | 0.062 |  | 1 | 3765 | EQ-5D-3L | Regression |  | NO | Canada |
| 229 | EQ-5D-derived health utilities and minimally important differences for chronic health conditions: 2011 Commonwealth Fund Survey of Sicker Adults in Canada | Kate Tsiplova | 0.027 | -0.002 | 0.056 |  | 1 | 1254 | EQ-5D-3L | Regression | Hypertension | NO | Canada |
| 230 | EQ-5D-derived health utilities and minimally important differences for chronic health conditions: 2011 Commonwealth Fund Survey of Sicker Adults in Canada | Kate Tsiplova | 0.014 | -0.036 | 0.064 |  | 1 | 455 | EQ-5D-3L | Regression | Heart Disease | NO | Canada |
| 231 | EQ-5D-derived health utilities and minimally important differences for chronic health conditions: 2011 Commonwealth Fund Survey of Sicker Adults in Canada | Kate Tsiplova | 0.020 | -0.038 | 0.078 |  | 1 | 568 | EQ-5D-3L | Regression | Diabetes Mellitus | NO | Canada |
| 232 | EQ-5D-derived health utilities and minimally important differences for chronic health conditions: 2011 Commonwealth Fund Survey of Sicker Adults in Canada | Kate Tsiplova | 0.028 | -0.007 | 0.063 |  | 1 | 1782 | EQ-5D-3L | Regression | Arthritis | NO | Canada |
| 233 | EQ-5D-derived health utilities and minimally important differences for chronic health conditions: 2011 Commonwealth Fund Survey of Sicker Adults in Canada | Kate Tsiplova | 0.035 | -0.010 | 0.080 |  | 1 | 614 | EQ-5D-3L | Regression | Asthma or Chronic Obstructive Pulmonary Disorder | NO | Canada |
| 234 | EQ-5D-derived health utilities and minimally important differences for chronic health conditions: 2011 Commonwealth Fund Survey of Sicker Adults in Canada | Kate Tsiplova | 0.012 | -0.038 | 0.062 |  | 1 | 716 | EQ-5D-3L | Regression | Anxiety or Depression | NO | Canada |
| 235 | EQ-5D-derived health utilities and minimally important differences for chronic health conditions: 2011 Commonwealth Fund Survey of Sicker Adults in Canada | Kate Tsiplova | 0.022 | -0.041 | 0.085 |  | 1 | 195 | EQ-5D-3L | Regression | Cancer | NO | Canada |
| 236 | EQ-5D-derived health utilities and minimally important differences for chronic health conditions: 2011 Commonwealth Fund Survey of Sicker Adults in Canada | Kate Tsiplova | 0.029 | -0.037 | 0.095 |  | 1 | 1054 | EQ-5D-3L | Regression | Chronic Back Pain | NO | Canada |
| 237 | Minimal Clinically Important Change for Pain Intensity, Functional Status, and General Health Status in Patients With Nonspecific Low Back Pain | Nicole van der Roer | 0.12 | -0.30 | 0.54 | 0.415 | 0 | 138 | EQ-5D-3L | Mean | Chronic Lower Back Pain | NO | Netherlands |
| 238 | Minimal Clinically Important Change for Pain Intensity, Functional Status, and General Health Status in Patients With Nonspecific Low Back Pain | Nicole van der Roer | 0.09 |  |  | 0.415 | 0 | 138 | EQ-5D-3L | ROC Curve | Chronic Lower Back Pain | NO | Netherlands |
| 239 | Minimal Clinically Important Change for Pain Intensity, Functional Status, and General Health Status in Patients With Nonspecific Low Back Pain | Nicole van der Roer | 0.19 | -0.33 | 0.71 | 0.415 | 0 | 304 | EQ-5D-3L | Mean | Sub-Acute Lower Back Pain | NO | Netherlands |
| 240 | Minimal Clinically Important Change for Pain Intensity, Functional Status, and General Health Status in Patients With Nonspecific Low Back Pain | Nicole van der Roer | 0.07 |  |  | 0.415 | 0 | 304 | EQ-5D-3L | ROC Curve | Sub-Acute Lower Back Pain | NO | Netherlands |
| 241 | Defining a Minimum Clinically Important Difference in Patient-Reported Outcome Measures in Lumbar Tubular Microdecompression Patients | Trent Vanhorn | 0.219 |  |  |  | 0 | 201 | EQ-5D-5L | ROC Curve | Symptomatic Lumbar Spinal Stenosis | YES | United States |
| 242 | What is the relationship between the minimally important difference and health state utility values? The case of the SF-6D | Stephen Walters | 0.039 | 0.034 | 0.044 | 0.357 | 1 | 4945 | SF-6D.v1 | Mean |  | NO | Great Britain |
| 243 | What is the relationship between the minimally important difference and health state utility values? The case of the SF-6D | Stephen Walters | 0.026 | 0.021 | 0.033 | 0.455 | 1 | 3127 | SF-6D.v1 | Mean |  | NO | Great Britain |
| 244 | Comparison of the minimally important difference for two health state utility measures: EQ-5D and SF-6D | Stephen Walters | 0.032 | -0.234 | 0.298 |  | 1 | 192 | SF-6D.v1 | Mean | Leg Ulcer | NO | Great Britain |
| 245 | Comparison of the minimally important difference for two health state utility measures: EQ-5D and SF-6D | Stephen Walters | 0.060 | -0.268 | 0.388 |  | 1 | 154 | SF-6D.v1 | Mean | Leg Ulcer | NO | Great Britain |
| 246 | Comparison of the minimally important difference for two health state utility measures: EQ-5D and SF-6D | Stephen Walters | 0.097 | -0.127 | 0.321 |  | 1 | 173 | SF-6D.v1 | Mean | Back Pain | NO | Great Britain |
| 247 | Comparison of the minimally important difference for two health state utility measures: EQ-5D and SF-6D | Stephen Walters | 0.037 | -0.187 | 0.261 |  | 1 | 244 | SF-6D.v1 | Mean | Early Rheumatoid Arthritis | NO | Great Britain |
| 248 | Comparison of the minimally important difference for two health state utility measures: EQ-5D and SF-6D | Stephen Walters | 0.038 | -0.196 | 0.272 |  | 1 | 57 | SF-6D.v1 | Mean | Limb Reconstruction | NO | Great Britain |
| 249 | Comparison of the minimally important difference for two health state utility measures: EQ-5D and SF-6D | Stephen Walters | 0.022 | -0.170 | 0.214 |  | 1 | 135 | SF-6D.v1 | Mean | Irritable Bowel Syndrome | NO | Great Britain |
| 250 | Comparison of the minimally important difference for two health state utility measures: EQ-5D and SF-6D | Stephen Walters | 0.025 | -0.211 | 0.261 |  | 1 | 173 | SF-6D.v1 | Mean |  | NO | Great Britain |
| 251 | Comparison of the minimally important difference for two health state utility measures: EQ-5D and SF-6D | Stephen Walters | 0.058 | -0.116 | 0.232 |  | 1 | 161 | SF-6D.v1 | Mean | Acute Myocardial Infarction | NO | Great Britain |
| 252 | Comparison of the minimally important difference for two health state utility measures: EQ-5D and SF-6D | Stephen Walters | 0.035 | -0.099 | 0.169 |  | 1 | 149 | SF-6D.v1 | Mean | Osteoarthritis of the Knee | NO | Great Britain |
| 253 | Comparison of the minimally important difference for two health state utility measures: EQ-5D and SF-6D | Stephen Walters | 0.139 | -0.527 | 0.805 |  | 1 | 192 | EQ-5D-3L | Mean | Leg Ulcer | NO | Great Britain |
| 254 | Comparison of the minimally important difference for two health state utility measures: EQ-5D and SF-6D | Stephen Walters | 0.109 | -0.697 | 0.915 |  | 1 | 154 | EQ-5D-3L | Mean | Leg Ulcer | NO | Great Britain |
| 255 | Comparison of the minimally important difference for two health state utility measures: EQ-5D and SF-6D | Stephen Walters | 0.081 | -0.557 | 0.719 |  | 1 | 173 | EQ-5D-3L | Mean | Back Pain | NO | Great Britain |
| 256 | Comparison of the minimally important difference for two health state utility measures: EQ-5D and SF-6D | Stephen Walters | 0.129 | -0.473 | 0.731 |  | 1 | 244 | EQ-5D-3L | Mean | Early Rheumatoid Arthritis | NO | Great Britain |
| 257 | Comparison of the minimally important difference for two health state utility measures: EQ-5D and SF-6D | Stephen Walters | 0.054 | -0.574 | 0.682 |  | 1 | 57 | EQ-5D-3L | Mean | Limb Reconstruction | NO | Great Britain |
| 258 | Comparison of the minimally important difference for two health state utility measures: EQ-5D and SF-6D | Stephen Walters | 0.065 | -0.427 | 0.557 |  | 1 | 135 | EQ-5D-3L | Mean | Irritable Bowel Syndrome | NO | Great Britain |
| 259 | Comparison of the minimally important difference for two health state utility measures: EQ-5D and SF-6D | Stephen Walters | 0.035 | -0.487 | 0.557 |  | 1 | 173 | EQ-5D-3L | Mean |  | NO | Great Britain |
| 260 | Comparison of the minimally important difference for two health state utility measures: EQ-5D and SF-6D | Stephen Walters | 0.089 | -0.395 | 0.573 |  | 1 | 161 | EQ-5D-3L | Mean | Acute Myocardial Infarction | NO | Great Britain |
| 261 | Comparison of the minimally important difference for two health state utility measures: EQ-5D and SF-6D | Stephen Walters | 0.121 | -0.519 | 0.761 |  | 1 | 149 | EQ-5D-3L | Mean | Osteoarthritis of the Knee | NO | Great Britain |
| 262 | Measuring the impact of chronic conditions and associated multimorbidity on health related quality of life in the general population in Hong Kong SAR, China: A cross-sectional study | Eliza Wong | 0.093 | 0.071 | 0.115 |  | 1 | 1014 | EQ-5D-5L | Instrument Defined | Various | NO | Hong Kong |
| 263 | Measuring the impact of chronic conditions and associated multimorbidity on health related quality of life in the general population in Hong Kong SAR, China: A cross-sectional study | Eliza Wong | 0.071 | 0.055 | 0.087 |  | 1 | 1014 | EQ-5D-5L | Instrument Defined | Various | NO | Hong Kong |
| 264 | Measuring the impact of chronic conditions and associated multimorbidity on health related quality of life in the general population in Hong Kong SAR, China: A cross-sectional study | Eliza Wong | 0.083 | 0.071 | 0.095 |  | 1 | 307 | EQ-5D-5L | Instrument Defined | Various | NO | Hong Kong |
| 265 | Measuring the impact of chronic conditions and associated multimorbidity on health related quality of life in the general population in Hong Kong SAR, China: A cross-sectional study | Eliza Wong | 0.079 | 0.067 | 0.091 |  | 1 | 307 | EQ-5D-5L | Instrument Defined | Various | NO | Hong Kong |
| 266 | Health‑related quality of life in elderly people with hypertension and the estimation of minimally important difference using EQ‑5D‑5L in Hong Kong SAR, China | Eliza Wong | 0.092 | 0.068 | 0.116 |  | 1 | 3351 | EQ-5D-5L | Instrument Defined | Hypertension | NO | Hong Kong |
| 267 | Health‑related quality of life in elderly people with hypertension and the estimation of minimally important difference using EQ‑5D‑5L in Hong Kong SAR, China | Eliza Wong | 0.072 | 0.055 | 0.088 |  | 1 | 3351 | EQ-5D-5L | Instrument Defined | Hypertension | NO | Hong Kong |
| 268 | Health‑related quality of life in elderly people with hypertension and the estimation of minimally important difference using EQ‑5D‑5L in Hong Kong SAR, China | Eliza Wong | 0.072 | 0.040 | 0.105 |  | 1 | 3351 | EQ-5D-5L | Instrument Defined | Hypertension | NO | Hong Kong |
| 269 | Health‑related quality of life in elderly people with hypertension and the estimation of minimally important difference using EQ‑5D‑5L in Hong Kong SAR, China | Eliza Wong | 0.070 | 0.036 | 0.104 |  | 1 | 3351 | EQ-5D-5L | Instrument Defined | Hypertension | NO | Hong Kong |
| 270 | Estimation of minimally important difference of the EQ-­5D-­5L utility scores among patients with either hypertension or diabetes or both: a cross-­sectional study in Hong Kong | Richard Xu | 0.089 | 0.065 | 0.113 |  | 1 | 847 | EQ-5D-5L | Instrument Defined | Hypertension and Diabetes | NO | Hong Kong |
| 271 | Estimation of minimally important difference of the EQ-­5D-­5L utility scores among patients with either hypertension or diabetes or both: a cross-­sectional study in Hong Kong | Richard Xu | 0.089 | 0.073 | 0.105 |  | 1 | 2231 | EQ-5D-5L | Instrument Defined | Hypertension | NO | Hong Kong |
| 272 | Estimation of minimally important difference of the EQ-­5D-­5L utility scores among patients with either hypertension or diabetes or both: a cross-­sectional study in Hong Kong | Richard Xu | 0.086 | 0.064 | 0.108 |  | 1 | 662 | EQ-5D-5L | Instrument Defined | Diabetes Mellitus | NO | Hong Kong |
| 273 | Estimation of minimally important difference of the EQ-­5D-­5L utility scores among patients with either hypertension or diabetes or both: a cross-­sectional study in Hong Kong | Richard Xu | 0.074 | 0.044 | 0.104 |  | 1 | 847 | EQ-5D-5L | Instrument Defined | Hypertension and Diabetes | NO | Hong Kong |
| 274 | Estimation of minimally important difference of the EQ-­5D-­5L utility scores among patients with either hypertension or diabetes or both: a cross-­sectional study in Hong Kong | Richard Xu | 0.073 | 0.043 | 0.103 |  | 1 | 2231 | EQ-5D-5L | Instrument Defined | Hypertension | NO | Hong Kong |
| 275 | Estimation of minimally important difference of the EQ-­5D-­5L utility scores among patients with either hypertension or diabetes or both: a cross-­sectional study in Hong Kong | Richard Xu | 0.073 | 0.049 | 0.097 |  | 1 | 662 | EQ-5D-5L | Instrument Defined | Diabetes Mellitus | NO | Hong Kong |
| 276 | Estimation of minimally important difference of the EQ-­5D-­5L utility scores among patients with either hypertension or diabetes or both: a cross-­sectional study in Hong Kong | Richard Xu | 0.069 | 0.041 | 0.097 |  | 1 | 847 | EQ-5D-5L | Instrument Defined | Hypertension and Diabetes | NO | Hong Kong |
| 277 | Estimation of minimally important difference of the EQ-­5D-­5L utility scores among patients with either hypertension or diabetes or both: a cross-­sectional study in Hong Kong | Richard Xu | 0.070 | 0.038 | 0.102 |  | 1 | 2231 | EQ-5D-5L | Instrument Defined | Hypertension | NO | Hong Kong |
| 278 | Estimation of minimally important difference of the EQ-­5D-­5L utility scores among patients with either hypertension or diabetes or both: a cross-­sectional study in Hong Kong | Richard Xu | 0.067 | 0.041 | 0.093 |  | 1 | 662 | EQ-5D-5L | Instrument Defined | Diabetes Mellitus | NO | Hong Kong |
| 279 | Estimation of minimally important difference of the EQ-­5D-­5L utility scores among patients with either hypertension or diabetes or both: a cross-­sectional study in Hong Kong | Richard Xu | 0.072 | 0.056 | 0.088 |  | 1 | 847 | EQ-5D-5L | Instrument Defined | Hypertension and Diabetes | NO | Hong Kong |
| 280 | Estimation of minimally important difference of the EQ-­5D-­5L utility scores among patients with either hypertension or diabetes or both: a cross-­sectional study in Hong Kong | Richard Xu | 0.071 | 0.055 | 0.087 |  | 1 | 2231 | EQ-5D-5L | Instrument Defined | Hypertension | NO | Hong Kong |
| 281 | Estimation of minimally important difference of the EQ-­5D-­5L utility scores among patients with either hypertension or diabetes or both: a cross-­sectional study in Hong Kong | Richard Xu | 0.071 | 0.055 | 0.087 |  | 1 | 662 | EQ-5D-5L | Instrument Defined | Diabetes Mellitus | NO | Hong Kong |
| 282 | Estimation of minimal clinically important change of the Japanese version of EQ-5D in patients with chronic noncancer pain: a retrospective research using real-world data | Kazetake Yoshizawa | 0.10 |  |  |  | 0 | 710 | EQ-5D-3L | ROC Curve | Chronic Pain | NO | Japan |

| **Section and Topic** | **Item #** | **Checklist item** | **Location where item**  **is reported** |
| --- | --- | --- | --- |
| **TITLE** | | |  |
| Title | 1 | Identify the report as a systematic review. | Page 1 |
| **ABSTRACT** | | |  |
| Abstract | 2 | See the PRISMA 2020 for Abstracts checklist. | Page 1 |
| **INTRODUCTION** | | |  |
| Rationale | 3 | Describe the rationale for the review in the context of existing knowledge. | Page 3 |
| Objectives | 4 | Provide an explicit statement of the objective(s) or question(s) the review addresses. | Page 3 |
| **METHODS** | | |  |
| Eligibility criteria | 5 | Specify the inclusion and exclusion criteria for the review and how studies were grouped for the syntheses. | Page 5, Page 7 |
| Information sources | 6 | Specify all databases, registers, websites, organisations, reference lists and other sources searched or consulted to identify studies. Specify the date when each source was last searched or consulted. | Page 5, Appendix A |
| Search strategy | 7 | Present the full search strategies for all databases, registers and websites, including any filters and limits used. | Page 5, Appendix A |
| Selection process | 8 | Specify the methods used to decide whether a study met the inclusion criteria of the review, including how many reviewers screened each record and each report retrieved, whether they worked independently, and if applicable, details of automation tools used in the process. | Page 5 |
| Data collection process | 9 | Specify the methods used to collect data from reports, including how many reviewers collected data from each report, whether they worked independently, any processes for obtaining or confirming data from study investigators, and if applicable, details of automation tools used in the process. | Page 5 |
| Data items | 10a | List and define all outcomes for which data were sought. Specify whether all results that were compatible with each outcome domain in each study were sought (e.g. for all measures, time points, analyses), and if not, the methods used to decide which results to collect. | Pages 5 and 6 |
|  | 10b | List and define all other variables for which data were sought (e.g. participant and intervention characteristics, funding sources). Describe any assumptions made about any missing or unclear information. | Pages 5 and 6 |
| Study risk of bias assessment | 11 | Specify the methods used to assess risk of bias in the included studies, including details of the tool(s) used, how many reviewers assessed each study and whether they worked independently, and if applicable, details of automation tools used in the process. | Pages 6 and 7 |
| Effect measures | 12 | Specify for each outcome the effect measure(s) (e.g. risk ratio, mean difference) used in the synthesis or presentation of results. | Page 7 |
| Synthesis methods | 13a | Describe the processes used to decide which studies were eligible for each synthesis (e.g. tabulating the study intervention characteristics and comparing against the planned groups for each synthesis (item #5)). | Page 8 |
|  | 13b | Describe any methods required to prepare the data for presentation or synthesis, such as handling of missing summary statistics, or data conversions. | NA |
|  | 13c | Describe any methods used to tabulate or visually display results of individual studies and syntheses. | Page 7 |
|  | 13d | Describe any methods used to synthesize results and provide a rationale for the choice(s). If meta-analysis was performed, describe the model(s), method(s) to identify the presence and extent of statistical heterogeneity, and software package(s) used. | Pages 7 and 8 |
|  | 13e | Describe any methods used to explore possible causes of heterogeneity among study results (e.g. subgroup analysis, meta-regression). | Pages 7 and 8 |
|  | 13f | Describe any sensitivity analyses conducted to assess robustness of the synthesized results. | NA |
| Reporting bias assessment | 14 | Describe any methods used to assess risk of bias due to missing results in a synthesis (arising from reporting biases). | NA |
| Certainty assessment | 15 | Describe any methods used to assess certainty (or confidence) in the body of evidence for an outcome. | Page 7 |

| **Section and Topic** | **Item #** | **Checklist item** | **Location where item**  **is reported** |
| --- | --- | --- | --- |
| **RESULTS** | | |  |
| Study selection | 16a | Describe the results of the search and selection process, from the number of records identified in the search to the number of studies included in the review, ideally using a flow diagram. | Pages 8 and 9, Figure 1 |
|  | 16b | Cite studies that might appear to meet the inclusion criteria, but which were excluded, and explain why they were excluded. | Page 8 |
| Study characteristics | 17 | Cite each included study and present its characteristics. | Pages 8-10, Supp. Table 1. |
| Risk of bias in studies | 18 | Present assessments of risk of bias for each included study. | Page 10, Table 2 |
| Results of individual studies | 19 | For all outcomes, present, for each study: (a) summary statistics for each group (where appropriate) and (b) an effect estimate and its precision (e.g. confidence/credible interval), ideally using structured tables or plots. | Pages 10-11, Tables 4 and 5, Supp Table 2. |
| Results of syntheses | 20a | For each synthesis, briefly summarise the characteristics and risk of bias among contributing studies. | NA |
|  | 20b | Present results of all statistical syntheses conducted. If meta-analysis was done, present for each the summary estimate and its precision (e.g. confidence/credible interval) and measures of statistical heterogeneity. If comparing groups, describe the direction of the effect. | NA |
|  | 20c | Present results of all investigations of possible causes of heterogeneity among study results. | NA |
|  | 20d | Present results of all sensitivity analyses conducted to assess the robustness of the synthesized results. | NA |
| Reporting biases | 21 | Present assessments of risk of bias due to missing results (arising from reporting biases) for each synthesis assessed. | NA |
| Certainty of evidence | 22 | Present assessments of certainty (or confidence) in the body of evidence for each outcome assessed. | Page 21 |
| **DISCUSSION** | | |  |
| Discussion | 23a | Provide a general interpretation of the results in the context of other evidence. | Pages 13-14 |
|  | 23b | Discuss any limitations of the evidence included in the review. | Pages 22-23 |
|  | 23c | Discuss any limitations of the review processes used. | Pages 22-23 |
|  | 23d | Discuss implications of the results for practice, policy, and future research. | Pages 13-14 and 23-24, Table 6 |
| **OTHER INFORMATION** | | |  |
| Registration and protocol | 24a | Provide registration information for the review, including register name and registration number, or state that the review was not registered. | 3 |
|  | 24b | Indicate where the review protocol can be accessed, or state that a protocol was not prepared. | 3-4 |
|  | 24c | Describe and explain any amendments to information provided at registration or in the protocol. | Pages 5-8 |
| Support | 25 | Describe sources of financial or non-financial support for the review, and the role of the funders or sponsors in the review. | Title Page |
| Competing interests | 26 | Declare any competing interests of review authors. | Title Page |
| Availability of data, code and other materials | 27 | Report which of the following are publicly available and where they can be found: template data collection forms; data extracted from included studies; data used for all analyses; analytic code; any other materials used in the review. | NA |

*From:* Page MJ, McKenzie JE, Bossuyt PM, Boutron I, Hoffmann TC, Mulrow CD, et al. The PRISMA 2020 statement: an updated guideline for reporting systematic reviews. BMJ 2021;372:n71. doi: 10.1136/bmj.n71

For more information, visit: <http://www.prisma-statement.org/>
